# Identification and validation of diagnostic markers and drugs for pediatric bronchopulmonary dysplasia based on integrating bioinformatics and molecular docking analysis

**DOI:** 10.1371/journal.pone.0323006

**Published:** 2025-05-07

**Authors:** Rui Guo, Qirui Zheng, Liang Zhang

**Affiliations:** 1 Neonatology, The First Affiliated Hospital of China Medical University, Shenyang, Liaoning Province, China; 2 Department of Ultrasound, The People’s Hospital of China Medical University, The People’s Hospital of Liaoning Province, Shenyang, Liaoning Province, China; University of Tennessee Health Science Center College of Medicine Memphis, UNITED STATES OF AMERICA

## Abstract

**Background:**

BPD is a prevalent chronic lung disease in infancy with lifelong impacts. Its early diagnosis and treatment are hindered by complex pathophysiology and limited mechanistic understanding. This study seeks to establish a foundation for early diagnosis and targeted therapy by identifying diagnostic markers and exploring drug-gene associations.

**Methods:**

Gene expression data were retrieved from the GEO database. Functional enrichment analyses were conducted on the differentially expressed genes (DEGs). DEGs were used to construct a PPI network. Three algorithms were applied to identify diagnostic markers. Immune cell infiltration was analyzed using the CIBERSORT tool, assessing relationships between immune cells and diagnostic markers. Molecular docking was performed to evaluate interactions between predict candidate drugs and diagnostic markers.

**Results:**

Six hub genes were identified as diagnostic markers. Diagnostic markers showed significant correlations with specific immune cells. Resveratrol and progesterone were found to stably bind to all six diagnostic markers in molecular docking analyses, suggesting therapeutic potential.

**Conclusion:**

In conclusion, our results show that IL7R, CXCL10, DEFA4, PRTN3, NCAPG and CCNB1 are BPD diagnostic indicators, and revealing immunological features associated with BPD. The molecular interactions of resveratrol and progesterone with the aforementioned key targets suggest their potential as therapeutic drugs for treating BPD.

## 1. Introduction

Bronchopulmonary dysplasia (BPD) is a major disease affecting the prognosis and quality of life of preterm infants. With advancements in perinatal medicine, the survival rates of preterm infants with lower gestational age and birth weight have increased in most countries, meanwhile the incidence of BPD has increased [1]. Previous studies have shown that BPD has lifelong impacts on adult health and quality of life, potentially leading to severe long-term pulmonary sequelae [2]. Follow-up studies of the BPD survivors suggested the lifelong consequences including compromised pulmonary function, asthma-like symptoms, pulmonary hypertension and exercise intolerance [3–6]. BPD imposes an increasingly significant burden on the healthcare system [7].

The pathophysiology of BPD involves a complex interplay of multiple factors and its prevention and lifelong consequences remain difficult to characterize. Therefore, it is important and valuable to identify diagnostic markers of BPD for improving diagnostic accuracy and guiding targeted therapies, which can enhance our understanding of the pathological development of BPD.

High-throughput sequencing and microarray have been extensively employed for the identification of potential genomic biomarkers and the analysis of gene expression changes within organisms, providing valuable insights for disease diagnosis and prognosis evaluation. Meanwhile, Machine learning (ML) algorithms have shown significant value in analyzing the potential relationships within high-dimensional data, which can be applied in the identification of biologically meaningful genes [8,9].

Therefore, our study analyzed high throughput sequencing data of BPD via integrating bioinformatics and performed Gene Ontology (GO), Kyoto Encyclopedia of Genes and Genomes (KEGG) analysis, Gene set enrichment analysis (GSEA) and protein-protein interaction (PPI) network analysis on the differentially expressed genes (DEGs) identified. Following that, we applied topological algorithms and ML algorithms to identify hub genes.

Moreover, considering the underlying crucial role of immune responses in the pathogenesis of BPD [10,11], our study also adopted immune cell infiltration analysis to investigate the relationship between diagnostic markers and immune cells, which can enhance our comprehension of the molecular immune mechanisms in BPD.

Currently, treatment options for BPD typically include surfactant, Glucocorticosteroids (GCS), caffeine, diuretics, inhaled bronchodilators, and Vitamin A, among others [12–16]. However, due to a lack of complete understanding of the mechanisms that lead to lung injury, which explains why these drugs are yielding satisfactory, ineffective, or even adverse outcomes under different circumstances [17]. It indicated that further studies are needed to study the potential therapeutic role of drugs on BPD and its detailed pharmacological mechanisms. Network pharmacology is a recent frontier in systematic drug research [18], and it can assist in evaluating the efficiency of multi-component, multi-target compound formulas and exploring more therapeutic strategies by targeting a specific network [19]. Compared with previous studies the present study utilized the network pharmacology and molecular docking approach to reveal the interactions between the treatment drugs and targets for the first time.

## 2. Materials and methods

### 2.1 Data collection from GEO and identification of DEGs

**Fig 1:**
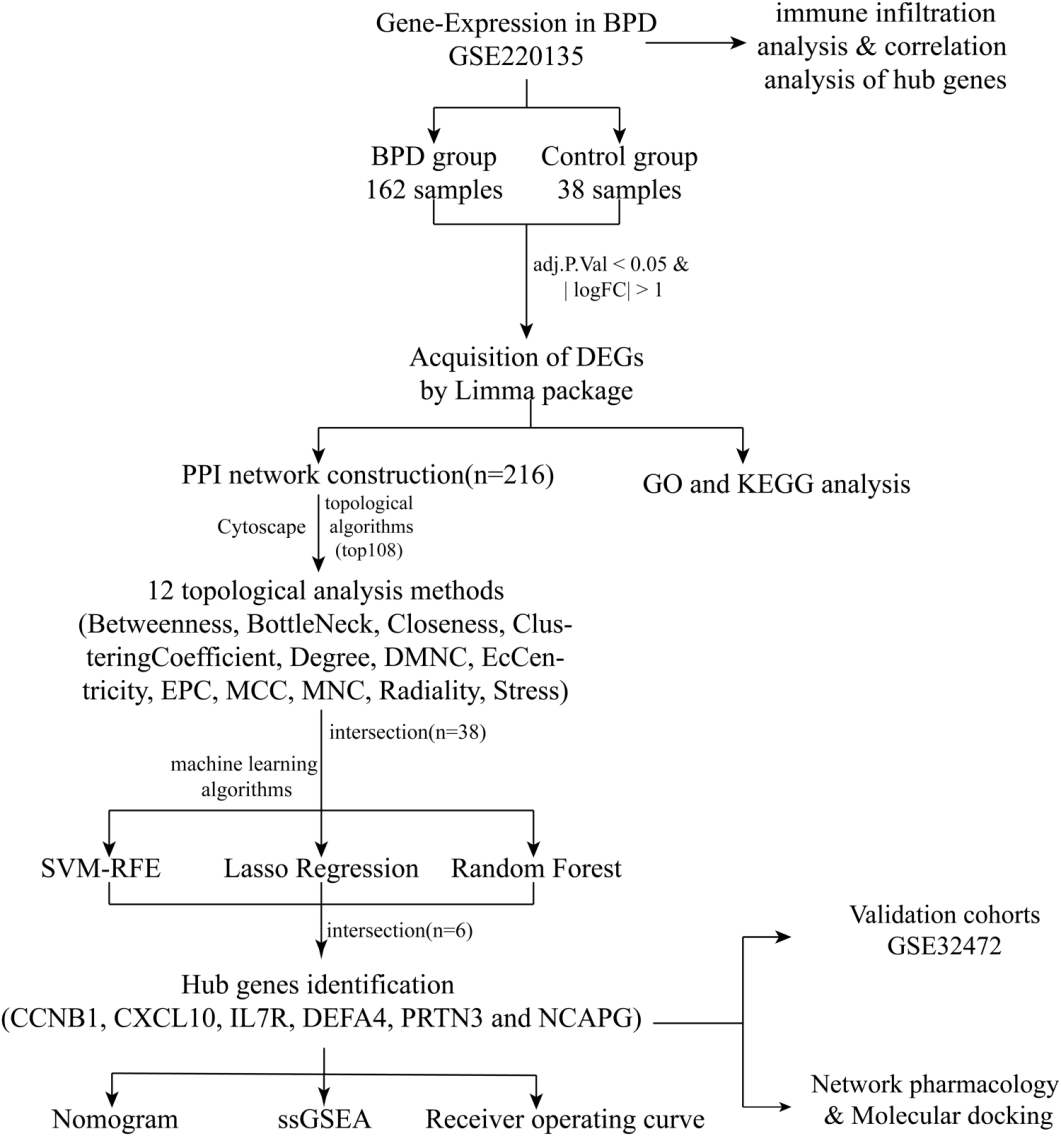
The study flowchart. Abbreviations:GEO, gene expression omnibus; BPD, Bronchopulmonary dysplasia; PPI, protein-protein interaction; GO, gene ontology; KEGG, Kyoto Encyclopedia of Genes and Genomes; DMNC, Maximum Neighborhood Component; EPC, EcCentricity, Edge Percolated Component; MCC, Maximal Clique Centrality; MNC, Maximum Neighborhood Component; SVM-RFE, Support Vector Machine-Recursive Feature Elimination; ssGSEA, Single-sample gene set enrichment analysis; CCNB1, Cyclin B1; CXCL10, the C-X-C motif chemokine 10; IL7R, IL-7 receptor; DEFA4, the Defensin Alpha4; PRTN3, Proteinase 3; NCAPG, non-SMC condensin I complex subunit G.

The study flowchart is depicted in Fig 1. The gene expression dataset were downloaded from the Gene Expression Omnibus (GEO, http://www.ncbi.nlm.nih.gov/geo/), it is a worldwide public database that features high-throughput microarray and next-generation sequencing functional genomic datasets that are submitted by the research community [20]. Finally, GSE220135 and GSE32472 were obtained using “BPD” as keyword and “Homo sapiens” as species. Dataset GSE220135 was set as the discovery cohort, and GSE32472 was utilized as the validation cohort. The GSE220135 [21] contains available 162 normal samples and 38 BPD samples. The GSE32472 [22] was divided into 112 no BPD samples as control samples and 42 serve BPD samples as BPD samples.

As for discovery cohort, the count matrix were read using R language. Then R package “DESeq2” was utilized to identify DEGs based on a threshold of | logFC| > 1.0 and an adjusted p-value < 0.05. The R package “ggplot2” was used to generate a volcano plot to visualize the identified DEGs.

### 2.2 Functional and pathway enrichment analyses

We used the online analysis tool DAVID (https://david.ncifcrf.gov/) to perform GO enrichment and KEGG pathway analyses based on DEGs and the adjusted p-value < 0.05 was set as the cut-off standard. The Gene Ontology (GO) knowledge base (http://geneontology.org) is a comprehensive resource concerning the functions of genes and gene products (proteins and non-coding RNAs) [23], and its terms contain biological process (BP), cellular component (CC), and molecular function (MF). The Kyoto Encyclopedia of Genes and Genomes (KEGG) is a manually curated database resource that integrates various biological objects, which are categorised into the following systems: systems, genomic, chemical and health information [24].

### 2.3 PPI network construction and pre-identifcation of hub genes

In order to enhance our comprehension of interactions among protein-coding genes, we utilized the Search Tool for the Retrieval of Interacting Genes (STRING) [25] online database (http://string-db.org) to construct a DEGs’s PPI network and it was imported into Cytoscape (version 3.7.2) [26]. In this network, we hid the nodes without connections with others and option the minimum required interaction score was 0.7. After that we used the plug-ins CytoHubba of Cytoscape and 12 topological analysis methods (Betweenness, BottleNeck, Closeness, Clustering Coefficient, Degree, Density of Maximum Neighborhood Component(DMNC), EcCentricity, Edge Percolated Component (EPC), Maximal Clique Centrality (MCC), Maximum Neighborhood Component (MNC), Radiality, Stress) to screened out hub genes. The overlapping DEGs of 12 topological algorithms were visualized by R packages “venn”. Furthermore, R packages “heatmap” was used to generate a heat map based on the overlapping DEGs.

### 2.4 Identification of diagnostic markers via ML algorithms

Support vector machine (SVM) is a supervised machine learning (ML) method capable of learning from data and making decisions [27]. Random Forest (RF) is a powerful feature selection, classification, and prediction method that offers several advantages, including no limitations on variable conditions and superior accuracy, sensitivity and specificity [28,29]. The least absolute shrinkage and selection operator (LASSO) is a data-mining method, which commonly used in multiple-linear regression to streamline models and provides widespread use in a variety of fields [30].

Therefore, we employed three machine learning algorithms to obtain diagnostic markers of BPD. SVMs was performed using the R package “e1071” and used 5-fold cross-validation to train the model to improve the performance of the model. RF was performed via the R package “randomForest”. LASSO regression analysis was performed using the R package “glmnet”.

### 2.5 Immune cell infiltration analysis

In order to estimate the relative abundance of each cell type in a bulk of cells from their gene expression profiles. We used the web tool CIBERSORT [31] (http://CIBERSORT.stanford.edu/) to calculate immune cell infiltration and explore the disease immune microenvironment. Then, we utilized histogram and boxplot to visualize the proportion of different immune cells in each sample and expression of difference immune cell between BPD group and control group respectively. Furthermore, Spearman correlation analysis was utilized to calculated the correlation of diagnostic markers and immune cells. The correlation between different immune cells was also analyzed. Only coefficients with P values below 0.05 will be included in the plots.

### 2.6 Single sample gene set enrichment analysis (ssGSEA)

ssGSEA is an extended version of gene set enrichment analysis that calculates an enrichment score for each sample-gene set pair. We conducted ssGSEA enrichment analyses using the signalling pathway dataset from the MSigDB (h.all.v2024.1.Hs.entrez.gmt) database [32] as the background. We subsequently utilized enrichplot to display the top 10 pathways based on normalized enrichment score (NES).

### 2.7 Receiver operating characteristic (ROC) evaluation and nomogram construction

We used GraphPad Prism (version 9.5) to established ROC curves to evaluate the diagnostic value of diagnostic markers for discovery cohort and validation cohort. We calculated the area under the ROC curve (AUC) and its 95% CI to quantify this value. An AUC value greater than 0.6 was considered an available diagnostic value. The R package “rms” was utilized to construct the nomogram based on logistic regression.

### 2.8 Network pharmacology and molecular docking

The combination of ML with network pharmacology is considered an important topic of biomedical sciences [19]. Enrichr is a comprehensive resource for curated gene sets and a search engine that accumulates biological knowledge for further biological discoveries [33]. We uploaded diagnostic markers to the Enrichr website (http://amp.pharm.mssm.edu/Enrichr) to predict therapeutic drugs using the DSigDB database. The protein structures of diagnostic markers were retrieved from Uniprot Data Bank (https://www.uniprot.org/) in PDB format. The 3D structures of candidate drugs were obtained from PubChem (http://pubchem.ncbi.nlm.nih.gov), which were transformed by Open Babel Toolkit (version 3.1.1) into a MOL2 file format. Finally, we used AutoDock software (version 1.5.7) to optimize protein structures for molecular docking and used Open-Source PyMOL (version 3.0.4) to visualize docking results.

### 2.9 Statistical analysis

If the comparison between two groups satisfies the equality of variance test, a t-test is used, otherwise a non-parametric test is employed, P-value < 0.05 was considered statistically significant. R software (version 4.4.1) and GraphPad Prism (version 9.5.0) were used to perform statistical analyses.

## 3. Results

### 3.1 Screening of DEGs and enrichment analysis

The research process is illustrated in Fig 1.

**Fig 2:**
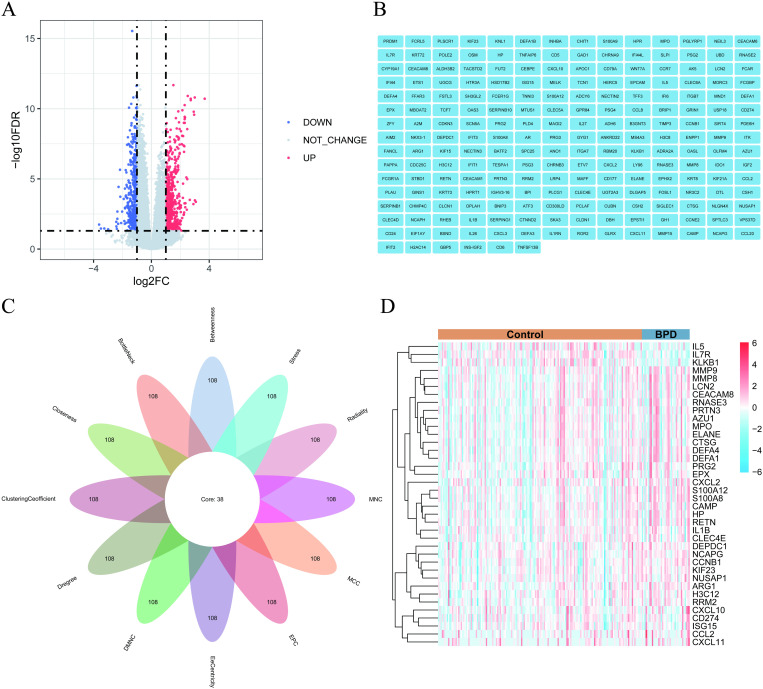
DEGs. (A) The volcano plot shows all DEGs, of which red and blue dots refer to significant DEGs. (B) Visualization of the PPI network genes by Cytoscape. (C) Venn diagram shows that 38 genes are identified from the Cytoscape using 12 topological analysis methods. (D) The heatmap displays the 38 upregulated and downregulated DEGs identified from Cytoscape, and each column represents one of BPD cases or controls. Red and blue represent upregulated and downregulated gene expression. DEGs differentially expressed genes.

**Fig 3:**
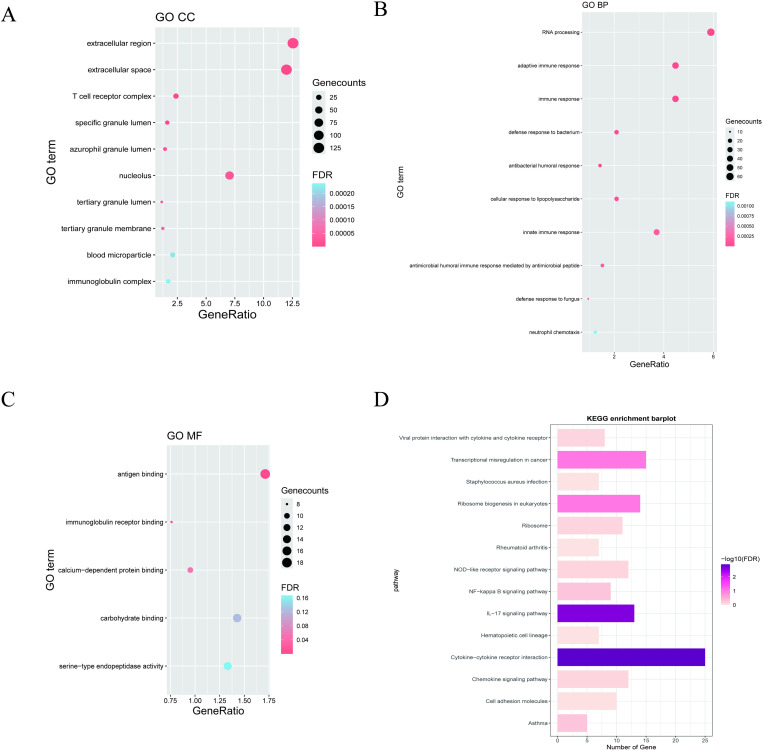
Enrichment analysis. (A-C) GO analysis of the DEGs, including biological process (BP), cellular component (CC), and molecular function (MF), respectively. The y-axis represents different GO terms, the x-axis represents gene ratio enriched in relative GO terms, the circle size refers to gene numbers, and the color represents p-value. (D) KEGG pathway analysis of the intersection of genes. Different colors represent various significant pathways and related enriched genes.

1103 DEGs were screened in discovery cohort with 615 upregulated and 488 downregulated genes (Fig 2A). Then we conducted GO enrichment and KEGG enrichment analysis on 1103 DEGs through the use of the DAVID online tool. The full results are presented in S1 Table. GO enrichment analysis showed: (1) CC: The DEGs were enriched in various locations, such as “extracellular region”, “extracellular space”, “T cell receptor complex”, “specific granule lumen”, “azurophil granule lumen” (Fig 3A). (2) BP: The DEGs were significantly enriched in several processes, including “RNA processing”, “adaptive immune response”, “immune response”, “defense response to bacterium”, “antibacterial humoral response” (Fig 3B). (3) MF: The DEGs were significantly enriched in “antigen binding” and “immunoglobulin receptor binding” (Fig 3C). Meanwhile, KEGG enrichment analysis demonstrated that the DEGs were significantly enriched in “cytokine-cytokine receptor interaction” and “IL-17 signaling pathway” metabolic pathways (Fig 3D).

### 3.2 PPI network construction and identification of diagnostic markers

All DEGs were uploaded to the STRING database to obtain PPI network, using high confidence (0.700) and the “hide disconnected nodes in network” as threshold. The PPI network containing 216 nodes was finally obtained (S1 Fig). To explore potential interactions between the DEGs, we imported the PPI network data attained into Cytoscape software (Fig 2B). We employed the Cytohubba plugin in Cytoscape to detect hub nodes and implemented 12 topological algorithms to calculate hub genes in the PPI network. After selecting the top 108 hub genes from the 12 algorithms (S2 Table), 38 overlapping hub genes were identified and displayed using a Venn diagram (Fig 2C), and the heat map based on 38 genes is presented in Fig 2D.

**Fig 4:**
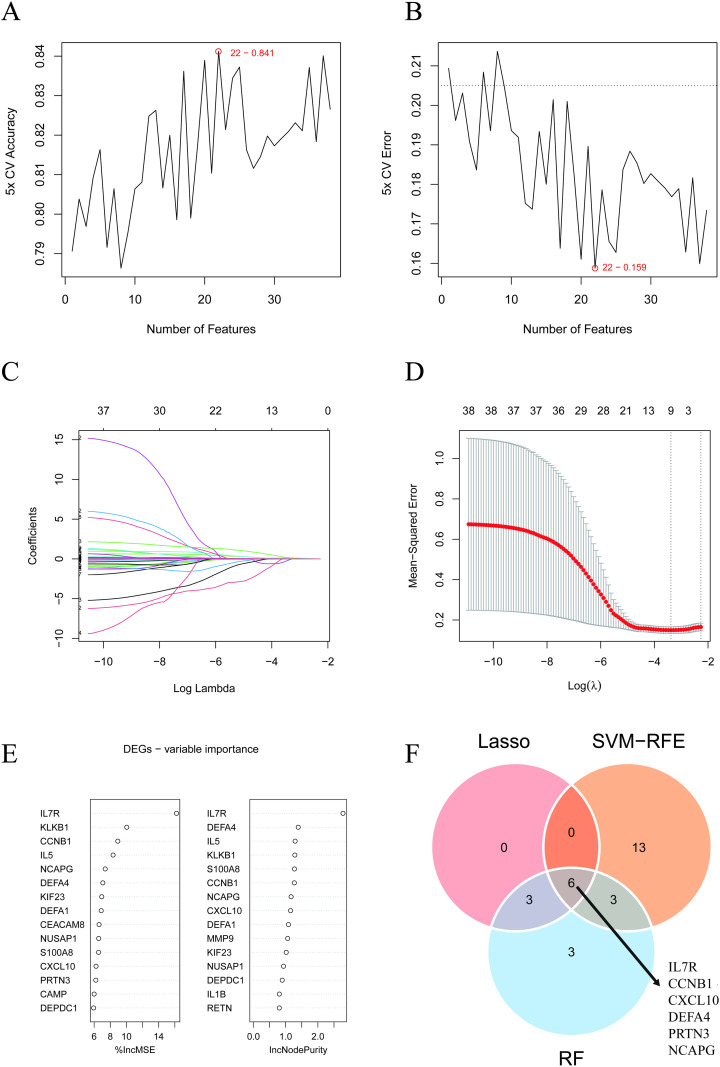
Machine learning. (A-B) Biomarkers was using the SVMs through 5-fold cross-validation. (C-D) LASSO logistic regression algorithm to screen diagnostic markers. (E) Based on RF algorithm to screen biomarkers. (F) Venn diagram shows that 6 genes are identified from three ML algorithms. SVM-RFE support vector machine-recursive feature elimination, RF random forest, LASSO least absolute shrinkage and selection operator. ML Machine Learning.

Following that, we employed three ML algorithms to identify diagnostic markers, and the 6 overlapping genes (CCNB1, CXCL10, IL7R, DEFA4, PRTN3, and NCAPG) were identified eventually which were visualized in Fig 4. Complete list of three ML algorithms was in S3 Table.

### 3.3 Immune cell infiltration

**Fig 5:**
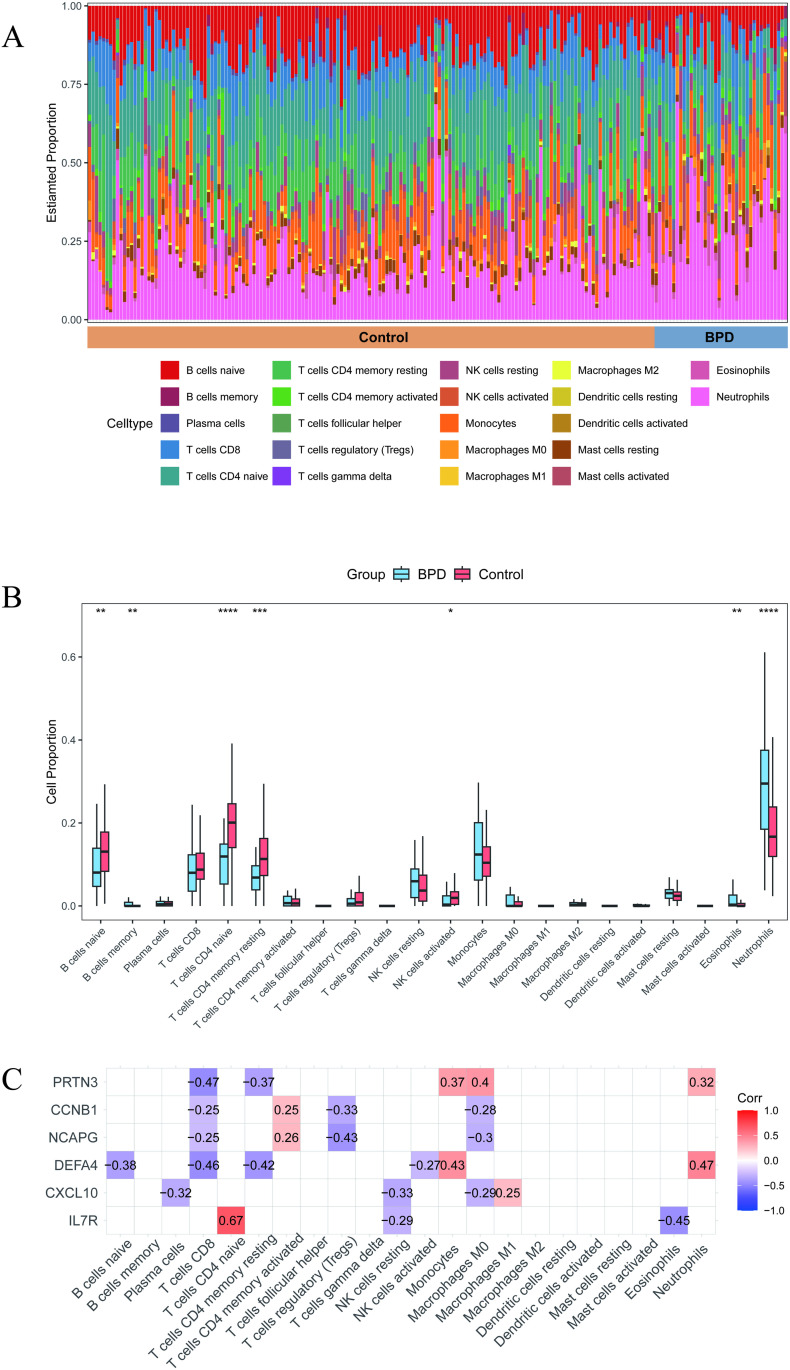
Immuno-infiltration analysis of the BPD dataset. (A) Histo-gram displays the results of 20 immune cell infiltrations. (B) Box plot displays the results differentially immune cell infiltrations between the BPD and non‐BPD group. An asterisk (*) signifies a P-value of < 0.05, indicating statistical significance. (C) Heat map displays the correlation analyses of immune infiltrated cells and immune infiltrated cells between six hub genes, red indicates a positive correlation, while blue represents a negative correlation.

We utilized CIBERSORT to quantify the proportions of 22 immune cell types, and each sample was shown as a staked bar plot (Fig 5A). The box plot (Fig 5B) revealed that neutrophils, B cells memory and eosinophils were higher in BPD group compared to control group. However, proportions of B cells naive, T cells CD4 naive, T cells CD4 memory resting and NK cells activated were lower in the BPD group compared to the control group. Moreover, the correlation analyses of immune infiltrated cells were investigated (S2 Fig). It shows that Dendritic cells activated had the top positive correlation with Macrophages M1 (r = 0.54), while neutrophils had the the largest negative correlation with T cells CD8 (r = -0.57) at same time.

Subsequently, we conducted correlation analyses between immune infiltrated cells and diagnostic markers (Fig 5C). In our analysis, IL7R showed a positive correlation with CD4 + naive T cells and a negative correlation with eosinophils, DEFA4 showed a positive correlation with neutrophils and a negative correlation with CD8 + T cells.

### 3.4 ssGSEA

**Fig 6:**
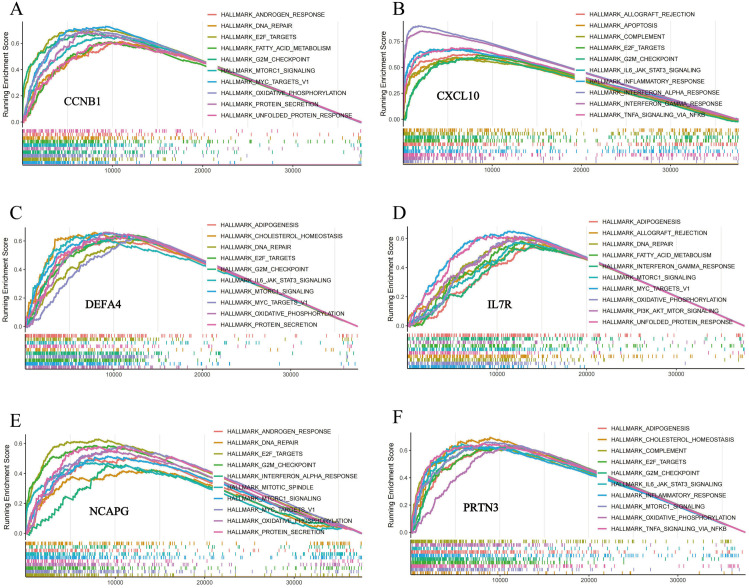
The enrichplot of ssGSEA for CCNB1, CXCL10, DEFA4, IL7R, NCAPG and PRTN3.

Each ssGSEA enrichment score indicates the degree of upregulation or downregulation of genes within a specific gene set in the sample (S4 Table). Based on the results, the top 10 pathways associated with the six genes as determined by NES were visualized (Fig 6). The results showed that all six hub genes were enriched in pathways such as myc targets v1, interferon gamma response and inflammation response.

### 3.5 Nomogram construction and ROC curves

**Fig 7:**
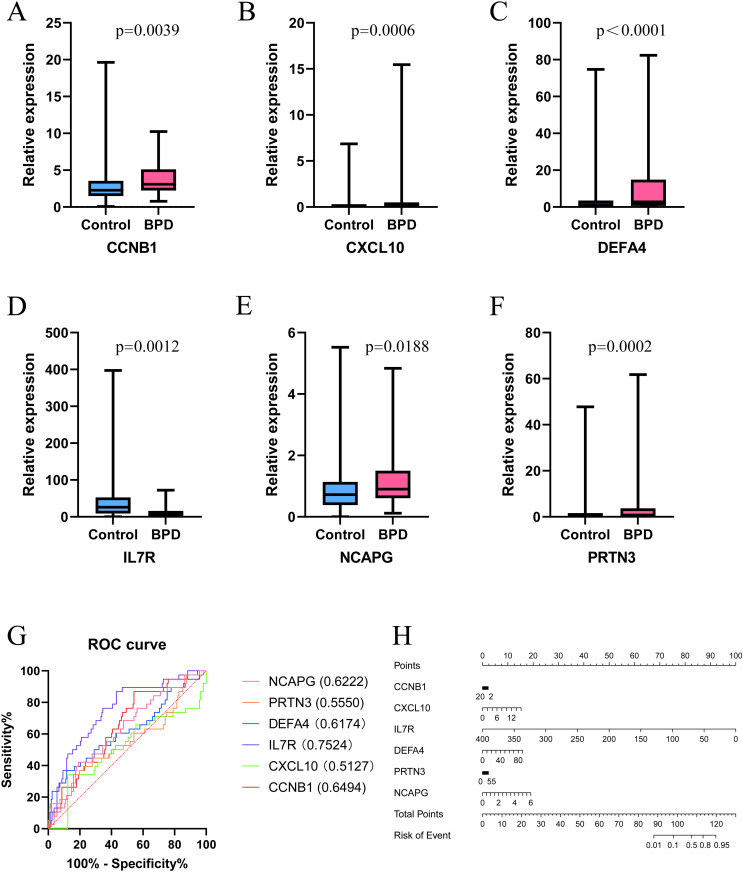
The diagnostic value evaluation and nomogram construction of the discovery cohort. (A-F)The Box plot showed expression of hub genes in BPDs and non-BPD groups. (G) The ROC curve of 6 hub genes in BPD. The number in the parentheses represents the AUC (Area Under the Curve).

Next, the nomogram showed that IL7R, CXCL10, and NCAPG had high predictive values (Fig 7H). Meanwhile, ROC curves were established for each of the six candidate hub genes to evaluate their diagnostic specificity and sensitivity (Fig 7G) and the results were as follows: IL7R (AUC 0.7524, CI 0.6689 0.8360), CCNB1 (AUC 0.6494, CI 0.5582–0.7407), CXCL10 (AUC 0.5127, CI 0.3982–0.6272), DEFA4 (AUC 0.6174, CI 0.5089–0.7260), PRTN3 (AUC 0.5550, CI 0.4440–0.6660) and NCAPG (AUC 0.6222, CI 0.5251–0.7192). The expression of hub genes was displayed using box plots generated in GraphPad Prism (Fig 7A–7F). Furthermore, the expression of five hub genes (IL7R, CXCL10, DEFA4, PRTN3, NCAPG) was verified in GSE32472 (S3 Fig).

### 3.6 Network pharmacology

**Fig 8:**
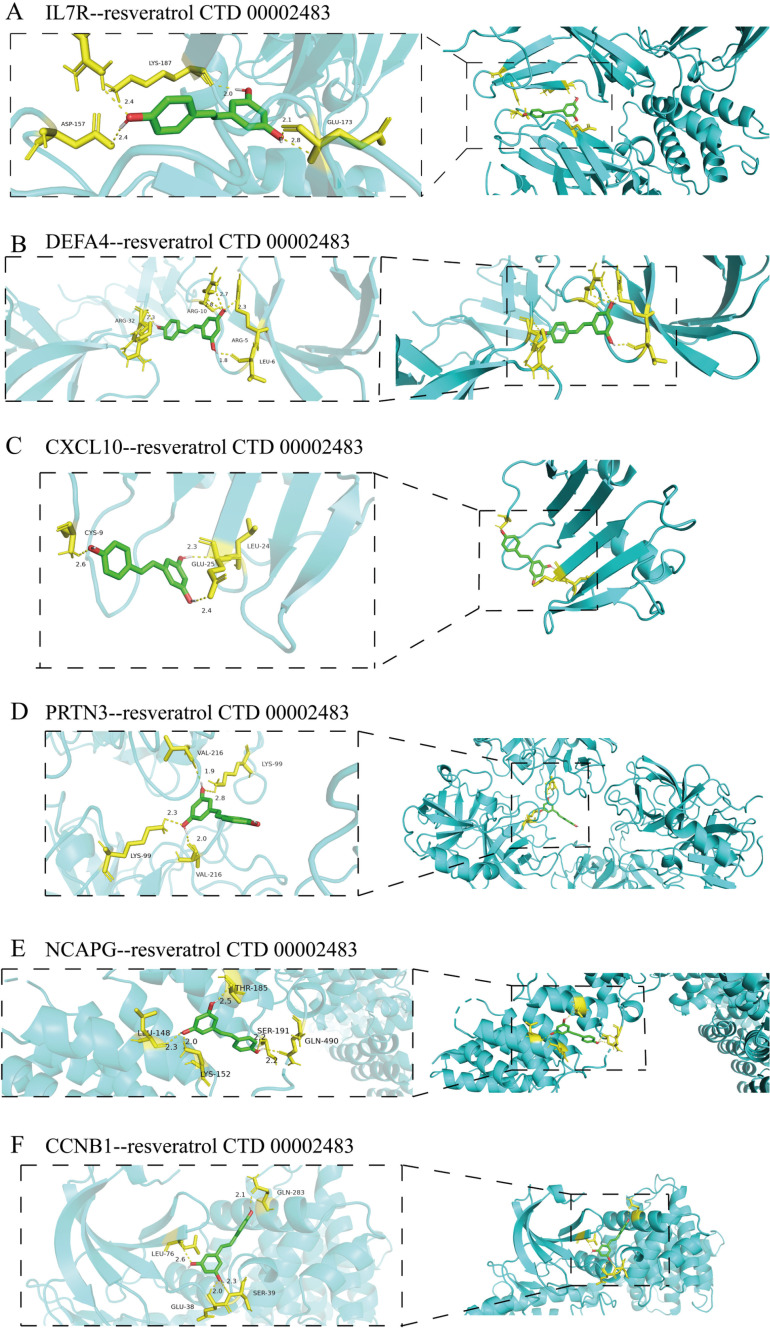
Molecular docking diagram of the six diagnostic bmarkers with resveratrol.

**Fig 9:**
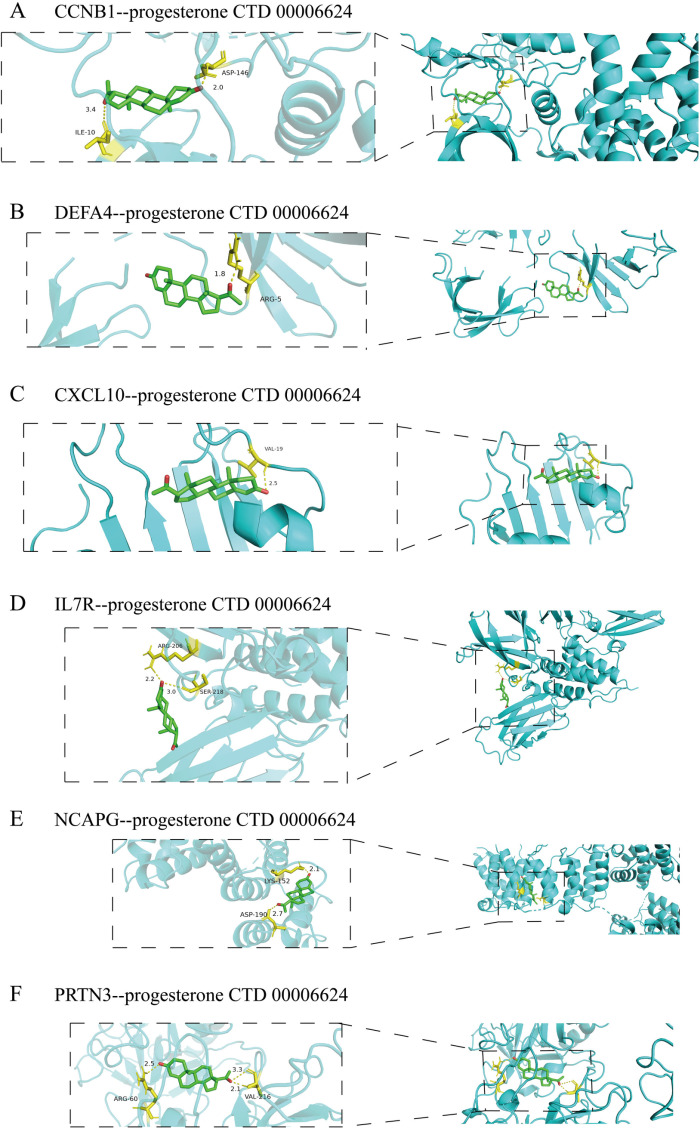
Molecular docking diagram of the six diagnostic bmarkers with progesterone.

We uploaded the six identified hub genes to the Enrichr website and obtained results for the candidate drug predicted using DSigDB (S5 Table). Based on the proportion of genes associated with predicted drugs and their safety profiles in humans, we ultimately selected resveratrol and progesterone as candidate drugs for further research. Next, we conducted molecular docking to identify potential binding between the drug and six diagnostic markers. All interactions between ligand-macromolecule complexes were mediated by hydrogen bonds, ensuring stability of these bindings. The result showed that both resveratrol (Fig 8) and progesterone (Fig 9) successfully bound to DEFA4, IL7R, CXCL10, PRTN3, CCNB1, and NCAPG, with docking energy values of: −5.49 kcal/mol, −5.20 kcal/mol, −3.76 kcal/mol, −5.00 kcal/mol, −5.07 kcal/mol, −4.76 kcal/mol and −8.83kcal/mol, −6.65kcal/mol, −7.72kcal/mol, −7.46kcal/mol, −8.39kcal/mol, −6.86kcal/mol. The smaller the docking energy value, the greater the stability of the binding between the drug and the gene.

## 4. Discussion

In this work, We not only attempted to identify diagnostic markers for BPD, but also utilized network pharmacology and molecular docking to reveal the interactions between predicted drugs and diagnostic markers. Meanwhile, we investigated the impact of immune cells on BPD and the relationship between diagnostic biomarkers and immune cells. In our study, Enrichment analysis revealed that the 1,103 differentially expressed genes we screened were mostly enriched in processes such as “adaptive immune response”, “T cell receptor complex”, and “immunoglobulin receptor binding”. Next, we further identified diagnostic markers by constructing a PPI network and using three ML algorithms. Ultimately, we identified DEFA4, IL7R, PRTN3, CXCL10, CCNB1, and NCAPG as the diagnostic markers for BPD.

IL 7 R regulates the activation and proliferation of immune cells through the activation of the JAK -STAT signaling pathways [34]. IL-7 receptor (IL-7R) is continually expressed by T cells in both the naive and memory states [35], and signaling through this receptor is essential for the long-term maintenance of all T cell populations. T lymphocytes play a crucial role not only in the recognition and elimination of pathogens, but also in coordinating tissue inflammatory responses and repair [36]. Alveolar macrophages (AMs) [37] and alveolar epithelial cells (AECs) [38] are crucial in the inflammatory response underlying lung inflammation. Studies have shown that certain drugs can successfully treat lung inflammation by blocking the IL-7/IL-7R pathways between macrophages and epithelial cells [39].

In addition to being a chemoattractant for different types of immune cells, human α-defensins can also induce the production of cytokines and chemokines [40]. Defensin Alpha 4 (DEFA4) is a member of the defensin family and is also known as Corticosterone Production (Corticostatin) [41] due to its antibacterial activity. Numerous studies have demonstrated that DEFA4 plays a crucial role not only in anti-microbial activity [42] and antiviral activity [43], but also in infectious diseases [43], and autoimmune diseases [44]. Furthermore, DEFA4 also plays a significant role in the respiratory system, and it is upregulated in various respiratory-related disorders, such as asthma [45] and idiopathic pulmonary fibrosis (IPF) [46].

In addition to binding to its receptor and triggering chemotaxis, cell growth, and apoptosis [47], the C-X-C motif chemokine 10 (CXCL10) also modulates immune response by recruiting inflammatory cells to the site of inflammation [48]. As an inflammation and macrophage-regulated chemokine [49], Loss of CXCL10 in BPD may restrict macrophage infiltration into the lungs, reduce lung apoptosis, attenuate pulmonary fibrotic remodeling in neonates, and promote alveolar growth [50].

Proteinase 3 (PR3) is a member of neutrophil serine proteases (NSPs) family [51] and it is encoded by the gene PRTN3 [52]. NSPs are considered to be multifunctional enzymes that participate in both pathogenic agent killing and regulation of inflammatory processes [53]. PR3 plays an important role in the anti-microbial activity of neutrophils and activation of neutrophils can result in the release of various cytotoxic products, leading to lung injury [54]. In addition to limiting lung development and contributing to BPD through the release of ROS, neutrophils can also disrupt the process of alveolar formation by releasing exosomes [55].

Cyclin B1 participates in mitochondrial dynamics [56] and Mitochondria-regulated apoptosis [57], which plays a significant role in cell cycle. When the CCNB1 gene is knocked out, the cell cycle is arrested [58]. Similarly, NCAPG, also known as non-SMC condensin I complex subunit G, not only leads to weakened cell migration and invasion ability after its knockout but also promotes cell apoptosis. However, our understanding of CCNB1 and NCAPG and their associated mechanisms in BPD is limited.

Although inflammation is a natural response to injury that aids in the healing process, it can also lead to further damage and dysfunction of the affected organ [59]. Inflammation plays a significant role in BPD and excessive inflammatory responses are the primary pathogenic mechanism of lung disease. We analyze the immune infiltration process in BPD to gain more comprehensive understanding of the impact of immune cell infiltration on the development of BPD. In our study, IL7R, DEFA4 and CXCL10 have been shown to be associated with various immune cells, and DEFA4 and CXCL10 were found to be upregulated, while IL7R was downregulated. which could offer new treatment avenues.

To further reveal the potential functions of these six hub genes, we conducted ssGSEA enrichment analysis. To test the predictive performance of the genes, we constructed nomogram and ROC curves and conducted external validation.

Subsequently, resveratrol and progesterone were selected as predicted drugs and subjected to molecular docking with the six aforementioned hub genes. Resveratrol (3,4’,5-trihydroxy-trans-stilbene) is a plant antitoxin that naturally exists in many dietary sources. It not only functions as an antioxidant by scavenging free radicals [60], but also possesses potential anti-inflammatory effects [61]. Studies have shown that resveratrol not only has anti-fibrotic effects [62], but also has a protective effect against neonatal oxygen-induced airway hyperreactivity [63]. The development of pharmaceutical formulations of resveratrol may become a prospective targeted treatment approach for BPD. Due to their potent anti-inflammatory effects, corticosteroids are utilized in the prevention or treatment of BPD, but their use can also increase the risk of neurological developmental disorders and other related diseases [64,65]. Both progesterone and corticosteroids belong to the class of steroid hormones, and progesterone is a critical participant in the interaction between the endocrine and immune systems [66], and further exploration is needed to determine whether progesterone can be used to treat BPD.

Interestingly, although the expression of 5 hub genes(IL7R, CXCL10, DEFA4, PRTN3, NCAPG) was validated in GSE32472, both of our predicted drugs successfully docked with 6 genes. In addition, while CXCL10 was upregulated in the discovery cohort, it was downregulated in the validation cohort, indicating the need for further research into the mechanisms of CXCL10 and CCNB1 in BPD.

The present study offers valuable insights into the potential of diagnostic markers and therapeutic agents for BPD. However, the study is not without its limitations. Firstly, the limited BPD sample size may reduce statistical robustness. Moreover, the analyses were predominantly based on transcriptomic data, with a paucity of integration with other histological data (e.g., proteomic, metabolomic, etc.). Furthermore, molecular docking results are typically based on static protein structures, which cannot fully model the dynamic behavior of proteins in vivo, necessitating further experimental validation, especially through in vitro and in vivo biological experiments. Finally, while the molecular docking results indicated that resveratrol and progesterone bind well to the diagnostic markers, the clinical development of these drugs remains challenging due to issues of bioavailability, toxicity, and safety for long-term use. It is imperative that these limitations are addressed in future studies by considering increasing the sample size, integrating multi-omics data, performing more comprehensive experimental validation, and further exploring the clinical translational potential of potential therapeutic agents.

## Supporting information

S1 Table GO and KEGG pathway enrichment analysis of overlapping DEGs(FDR < 0.05). (DOCX)

S2 Table Complete list of Top108 DEGs from Degree,Closeness and Betweenness algorithms via CytoHubba plug-in. (DOCX)

S3 Table Complete list of three machine learning algorithms. (DOCX)

S4 Table Complete list of ssGSEA results (adj.P value <0.05). (DOCX)

S5 Table Candidate drug predicted using DSigDB (adj.P value <0.05). (DOCX)

S1 Fig The PPI network via STRING database. (DOCX)

S2 Fig The correlated heatmap showed the correlation analysis of immune infiltrated cells. (DOCX)

S3 Fig The diagnostic value evaluation and nomogram construction of the validation cohort. (DOCX)
