# Supplementary material for: Identification and validation of diagnostic markers and drugs for pediatric bronchopulmonary dysplasia based on integrating bioinformatics and molecular docking analysis
Source: PLoS One. 2025 May 7;20(5):e0323006. doi: 10.1371/journal.pone.0323006 (PMC12057968; doi:10.1371/journal.pone.0323006)
Supplement: S1 Table — (DOCX) [file pone.0323006.s001.docx]

S1 Table. GO and KEGG pathway enrichment analysis of overlapping DEGs(FDR<0.05)

| GO ID | Description | Count | Genes |
| --- | --- | --- | --- |
| Biological Process | | | |
| GO:0006396 | RNA processing | 62 | SNORA84, SNORA14B, SCARNA1, SNORD113-4, SNORA3A, SNORD49A, SNORA38, MIR3651, SNORA73A, SNORA78, SNORA73B, SNORA79, SNORA70, SNORA59B, SNORA79B, SNORA2C, SNORA2A, MIR6516, SNORA27, SNORA10B, SNORA67, SNORA68, SNORA50A, SNORA21, SNORA22, SNORA50C, SNORA63, SNORA64, SNORA20, SCARNA14, SNORA16A, SNORA36A, SNORA36B, SNORD3D, SCARNA18, SCARNA16, SNORA15, SNORA31B, SNORA12, SNORA71A, SNORA54, SNORA10, SNORA63E, SNORA52, SNORA71C, SNORA53, SNORA71D, SNORA17B, SCARNA22, SCARNA20, SNORD88C, SNORA77B, MIR664B, MIR664A, SCARNA8, SNORA80D, SNORA49, MIR1248, SNORA48, SNORA40B, SNORA80A, SNORA80B |
| GO:0002250 | adaptive immune response | 47 | CD274, TRBV17, IGHM, ITK, TRBV10-2, TRBV19, TRBV4-1, TRAV22, TRBV11-3, TRBV7-6, TRBV6-7, TRBV6-8, TRBV16, IGLV4-60, IGHG3, CD79A, IGHG4, IGHG1, IGHG2, TRAV23DV6, LAMP3, IGKV1-13, TRAV38-1, IGHA1, TRBV20-1, TRBV23-1, IGKV1-8, ARG1, TRDV1, TRBJ2-6, TRAJ3, TRBJ2-5, TRBJ2-7, BMX, TRBJ2-4, TRBJ1-5, IGKV1D-13, TRBJ1-1, CLEC4D, CD6, CLEC6A, IGLV3-12, IGHJ1, TARM1, TRAV8-4, TRAV26-2, TRAJ31 |
| GO:0006955 | immune response | 47 | FCGR1BP, CD274, IL1RN, TCF7, IGHV4-34, IFI6, IGHV4-39, CST7, ETS1, IFI44L, FCAR, IGLV4-60, TNFSF13B, IL36A, CCL2, IGKV1-13, ENPP1, CCR7, CTSG, DEFA1B, FCGR1A, TRAV38-1, IGHA1, IGHV3-30, IGHV3-53, IGKV1-8, FCRL5, CCL20, IGHV3-33, TRDV1, OSM, PRG2, IFI44, DEFA1, PRG3, IGKV1D-13, CHIT1, FCER2, RAET1E, AIM2, IL5, SLPI, IL1B, IGLV3-12, TRAV8-4, CEACAM8, IL7R |
| GO:0042742 | defense response to bacterium | 22 | GBP5, FCER1G, ANXA3, CCL20, HP, PRG2, ISG15, LPO, HPR, EPX, MPO, LYZL6, CLEC4D, OAS3, LCN2, S100A12, FCGR1A, CLEC4E, S100A9, S100A8, ELANE, CAMP |
| GO:0019731 | antibacterial humoral response | 15 | IGHM, DEFA4, DEFA3, DEFA1, RNASE3, AZU1, IGHG3, IGHG4, IGHG1, IGHG2, SLPI, CTSG, DEFA1B, IGHA1, CAMP |
| GO:0071222 | cellular response to lipopolysaccharide | 22 | GBP5, CD274, CEBPE, DEFA4, ACOD1, DEFA3, LY96, DEFA1, AZU1, CXCL3, MMP8, CXCL2, MMP9, FCAR, CXCL10, CXCL11, IL36A, IL1B, CCL2, CTSG, DEFA1B, CAMP |
| GO:0045087 | innate immune response | 39 | IGHM, CRISP3, IFI6, LY96, PLD4, IL27, INAVA, LILRA5, OASL, HERC5, IL36A, CLEC5A, SUSD4, S100A12, FCGR1A, PGLYRP1, CAMP, CD177, GBP5, FCER1G, ARG1, DEFA4, TRDV1, ISG15, RNASE3, AIM2, VNN1, CD6, SLPI, HKDC1, OAS3, CLEC6A, TARM1, LCN2, SERPING1, BPI, CLEC4E, S100A9, S100A8 |
| GO:0061844 | antimicrobial humoral immune response mediated by antimicrobial peptide | 16 | CCL20, DEFA4, DEFA3, DEFA1, RNASE3, CXCL3, CXCL2, CXCL10, CXCL11, CCL8, S100A12, CCL2, DEFA1B, PGLYRP1, S100A9, CAMP |
| GO:0050832 | defense response to fungus | 10 | DEFA4, CLEC6A, DEFA3, S100A12, CTSG, DEFA1, DEFA1B, MPO, S100A9, S100A8 |
| GO:0030593 | neutrophil chemotaxis | 13 | FCER1G, CCL20, CXCL3, CXCL2, CXCL10, CXCL11, CCL8, IL1B, DPEP1, S100A12, CCL2, S100A9, S100A8 |
| GO:0006954 | inflammatory response | 31 | TNFAIP6, LY96, PLD4, IL27, CXCL3, CXCL2, CCL8, S100A12, CCL2, OLR1, FFAR3, CCR7, GBP5, PLA2G2D, CCL20, ACOD1, AZU1, FOSL1, CXCL10, CXCL11, AIM2, VNN1, IL5, ELF3, HKDC1, IL1B, DPEP1, SIGLEC1, S100A9, S100A8, IDO1 |
| GO:0007166 | cell surface receptor signaling pathway | 25 | FCGR1BP, CD274, TRBV17, TRBV10-2, TRBV19, TRBV4-1, LY96, TRBV11-3, TRBV7-6, TRBV6-7, TRBV6-8, TRBV16, ADGRG7, CCL2, MS4A12, FCGR1A, TRBV20-1, TRBV23-1, FCRL5, INHBA, CXCL10, TRBV20OR9-2, PAPPA, ADGRD2, IL7R |
| GO:0006935 | chemotaxis | 14 | ROBO3, CCL20, DEFA1, RNASE2CP, RNASE3, RNASE2, CXCL2, FOSL1, CXCL10, CXCL11, CCL8, PLAU, CCL2, DEFA1B |
| GO:0016064 | immunoglobulin mediated immune response | 13 | IGHM, IGHV3-73, IGHV3-30, FCER1G, IGHV3-53, IGHV3-33, IGHV4-34, IGHV4-39, IGHV1-24, IGHV2-70D, IGHV3-49, IGHV3-16, IGHV3-64D |
| GO:0002227 | innate immune response in mucosa | 8 | DEFA4, DEFA3, DEFA1, RNASE3, RNASE2CP, DEFA1B, RNASE2, CAMP |
| GO:0000353 | formation of quadruple SL/U4/U5/U6 snRNP | 5 | RNU4-1, RNU5F-1, RNU5E-1, RNU5A-1, RNU4-2 |
| GO:0051607 | defense response to virus | 19 | GBP5, BNIP3, IFI6, ACOD1, DEFA3, ISG15, DEFA1, AZU1, IFIT1, RNASE2, IFIT3, IFI44L, IFIT2, OASL, HERC5, PLSCR1, AIM2, OAS3, DEFA1B |
| GO:0050853 | B cell receptor signaling pathway | 9 | IGHG3, IGHM, ITK, CD79A, IGHG4, IGHG1, IGHG2, BMX, IGHA1 |
| Cellular Component | | | |
| GO:0005576 | extracellular region | 132 | HP, IGHV2-70D, EPX, MPO, IGHG3, IGHG4, IGHG1, IL36A, IGHG2, PLAU, ENPP1, LIPM, NRCAM, SVEP1, PGLYRP1, GYG1, IGHV3-30, SPINK8, IGHV3-33, PSG3, PSG2, RNASE3, OLFM4, RNASE2, EREG, CHIT1, SLPI, PSG4, PAPPA, UTS2, SERPING1, SPARCL1, PRTN3, S100A9, S100A8, COL17A1, LCN8, CSH2, HTRA3, CSH1, NXPH4, LY96, LPO, HPR, LYPD6, TMEM25, S100A12, NPTX1, A2M, IGHA1, ELANE, COL28A1, NTNG2, DEFA4, IGF2, PRG2, DEFA3, ISG15, IGHV3-16, INHBA, AZU1, DEFA1, PRG3, HRNR, CXCL10, GH1, CXCL11, VNN1, IL5, TCN1, COL5A2, LCN2, IL7R, PSCA, TNFAIP6, IL26, CRISP3, NCAN, PZP, IGHV4-34, IGHV4-39, IL27, DBH, CXCL3, CXCL2, FCAR, TNFSF13B, OLR1, TIMP3, CTSG, CAMP, SRGN, H3C8, SERPINB1, IGHV3-73, ARG1, TMPRSS11E, MMP8, MMP9, IL1B, IGHJ1, TFF3, BPI, IGHV1-24, IGHV3-49, RETN, FSTL3, LYZL6, LILRA5, APOL4, SPOCK2, ADAM23, H3C12, SUSD4, CCL2, DEFA1B, IGHV3-64D, PLA2G2D, IGHV3-53, GCA, GDF15, CCL20, OSM, WNT7A, PLXDC1, KISS1, CD6, APOC1, S100P, SIGLEC1, KLKB1, SIGLEC6 |
| GO:0005615 | extracellular space | 126 | FCGBP, SPON1, IL1RN, HP, MRGPRD, EPX, MPO, IGHG3, IGHG4, IGHG1, IL36A, IGHG2, PLAU, IGKV1-13, ENPP1, SVEP1, PGLYRP1, TRAV38-1, IGHV3-30, KRT8, MTUS1, RNASE3, OLFM4, RNASE2, KRT73, KRT72, EREG, CHIT1, MDS2, SLPI, PAPPA, DPEP1, UTS2, SERPING1, SPARCL1, PRTN3, S100A9, S100A8, COL17A1, CSH2, CSH1, CHRD, TACSTD2, LPO, HPR, S100A12, A2M, IGHA1, ELANE, COL28A1, DEFA4, TFPI2, TRDV1, IGF2, DEFA3, INHBA, AZU1, DEFA1, IGKV1D-13, CXCL10, GH1, BMP3, CXCL11, RAET1E, IL5, INS-IGF2, TCN1, COL5A2, LCN2, IGHM, TNFAIP6, IL26, CRISP3, NCAN, PZP, WFDC1, IL27, DBH, LRRC32, CXCL3, CXCL2, IGLV4-60, TNFSF13B, S100A7A, TIMP3, CTSG, CAMP, SRGN, SERPINB1, ARG1, MMP8, MMP9, CEACAM6, MMP15, OAS3, IL1B, IGLV3-12, TFF3, BPI, CEACAM8, SERPINB10, KRT81, RNASE2CP, RETN, CST7, FSTL3, LILRA5, APOL4, CCL8, SPOCK2, PRRG1, CCL2, CILP2, DEFA1B, LINGO1, IGKV1-8, GDF15, CCL20, OSM, WNT7A, PLXDC1, MIR664A, KISS1, CD6, ERVH48-1, KLKB1 |
| GO:0042101 | T cell receptor complex | 25 | TRBV17, TRBV10-2, TRBV4-1, TRAV22, TRBV11-3, TRBV7-6, TRBV6-7, TRBV6-8, TRBV16, TRAV23DV6, TRAV38-1, TRBV20-1, TRBV23-1, TRDV1, TRBJ2-6, TRAJ3, TRBJ2-5, TRBJ2-7, TRBJ2-4, TRBJ1-5, TRBJ1-1, CEACAM1, CD6, TRAV8-4, TRAV26-2 |
| GO:0035580 | specific granule lumen | 17 | ARG1, DEFA4, CRISP3, HP, HPR, RETN, PRG3, OLFM4, MMP8, CHIT1, SLPI, TCN1, LCN2, BPI, PGLYRP1, ELANE, CAMP |
| GO:0035578 | azurophil granule lumen | 15 | GCA, ARG1, DEFA3, DEFA1, RNASE3, AZU1, RETN, RNASE2, MPO, HRNR, CTSG, PRTN3, BPI, DEFA1B, ELANE |
| GO:0005730 | nucleolus | 74 | SNORA84, SNORA14B, PRDM5, SCARNA1, SNORD113-4, SNORA3A, SNORD49A, OASL, SNORA38, NUSAP1, MIR3651, SNORA73A, SNORA78, SNORA73B, SCN5A, SNORA79, VCX3B, SNORA70, SNORA59B, SNORA79B, MTUS1, SNORA2C, SNORA2A, ZFY, PLSCR1, MIR6516, SNORA27, SNORA10B, SNORA67, SNORA68, SNORA50A, SNORA21, SNORA22, DTL, SNORA50C, SNORA63, ATF3, SNORA64, SNORA20, SCARNA14, SNORA16A, SNORA36A, SNORA36B, SNORD3D, SCARNA18, SCARNA16, SNORA15, SNORA31B, SNORA12, C19ORF33, SNORA71A, SNORA54, SNORA10, SNORA63E, SNORA52, SNORA71C, SNORA53, SNORA71D, SNORA17B, SCARNA22, SCARNA20, SNORD88C, SNORA77B, MIR664B, MIR664A, SCARNA8, SNORA80D, LIN28B, SNORA49, MIR1248, SNORA48, SNORA40B, SNORA80A, SNORA80B |
| GO:1904724 | tertiary granule lumen | 12 | CHIT1, TNFAIP6, TCN1, CRISP3, HP, HPR, PRG3, PGLYRP1, OLFM4, MMP8, MMP9, CAMP |
| GO:0070821 | tertiary granule membrane | 13 | FCER1G, MCEMP1, GPR84, FCAR, CEACAM1, CLEC4D, PLAU, STBD1, TARM1, CLEC5A, OLR1, CEACAM8, CD177 |
| GO:0072562 | blood microparticle | 22 | IGHM, IGHV3-73, IGHV3-30, IGHV3-53, IGHV3-33, PZP, IGHV4-34, HP, IGHV4-39, IGHV1-24, IGHV2-70D, IGHV3-49, HPR, IGHV3-16, IGHG3, IGHG4, IGHG1, IGHG2, SERPING1, A2M, IGHA1, IGHV3-64D |
| GO:0019814 | immunoglobulin complex | 18 | IGHM, IGHV3-73, IGHV3-30, IGHV3-53, IGKV1-8, IGHV3-33, IGHV4-34, IGHV4-39, IGHV1-24, IGHV2-70D, IGHV3-49, IGHV3-16, IGKV1D-13, IGLV4-60, IGLV3-12, IGHJ1, IGKV1-13, IGHV3-64D |
| GO:0035579 | specific granule membrane | 13 | MS4A3, MCEMP1, GPR84, SLC2A5, FCAR, CEACAM1, CLEC4D, PLAU, TARM1, CLEC5A, OLR1, CEACAM8, CD177 |
| GO:0005886 | plasma membrane | 207 | IL1RN, TTLL10, IGHV2-70D, MRGPRD, ABCA13, CLDN1, GJA4, IGKV1-13, OR2A42, MS4A12, CUBN, EPHA4, IGHV3-30, IGHV3-33, MTUS1, PSG3, MCEMP1, PSG2, SLC51A, EREG, AR, PSG4, GPR160, TRAJ31, HPN, SLCO5A1, LYPD6, CD79A, GNG10, GALR1, S100A12, C19ORF33, PLCG1, IGHA1, NTNG2, IGHV3-16, RAET1E, VNN1, OR6A2, XKR5, OR2A1, ITK, ROBO3, IGHM, SLC44A3, DOC2B, LRRC32, TRBV6-7, JPH1, TRBV6-8, MS4A4A, STBD1, FFAR3, OLR1, TNFAIP8L3, CCR7, CTSG, FCER1G, ANXA3, SYN2, RGMA, FCER2, ANO1, CEACAM1, PLSCR1, CEACAM6, MMP15, IGLV3-12, B3GNT3, VN1R1, CEACAM8, SERPINB10, ATP23, IFI6, GPR42, IGHV1-24, TRBV7-6, ADCY6, LILRA5, SLC7A3, EPCAM, GPA33, PERP, ADAM23, IGHV3-64D, PAK3, TRBV23-1, GCA, IGKV1-8, GAD1, WNT7A, ADAM32, PLXDC1, GRIN1, TRBV20OR9-2, TRAV8-4, SIGLEC1, KLKB1, SIGLEC6, SLC28A3, TRBV17, PHLDB1, TRBV19, AMHR2, TRBV4-1, CTNND2, RTKN2, TRBV11-3, TRBV16, IGHG3, IGHG4, IGHG1, IGHG2, PLAU, LAMP3, CLEC5A, ENPP1, SVEP1, NRCAM, TGM5, SH3GL2, CD177, SEMA6B, CHRNB3, SCARA5, KCNH8, MAGI2, OLFM4, EDAR, OR2T3, MELK, CLEC4D, CHMP4C, DPEP1, PRTN3, ROR2, CLEC4E, S100A9, AJUBA, S100A8, COL17A1, CD274, TRBV10-2, GPR88, CHRNA9, LY96, SLC1A3, GPR84, CACNA1E, TMEM25, ADGRG7, CD300LD, CCDC141, OR5B21, BMX, IGKV1D-13, TRPV6, RHEB, CLEC6A, TRPV4, TARM1, SLC26A8, IL7R, FCGR1BP, MS4A3, PSCA, IGHV4-34, IGHV4-39, SLC2A5, FCAR, IGLV4-60, TNFSF13B, PSTPIP2, ITGB7, SCN5A, SCARF1, TAS2R4, TRBV20-1, IGHV3-73, HTR3A, TMPRSS11E, ADRA2A, OAS3, IGHJ1, CDHR3, ITGA7, OR10A2, CEBPE, LRP4, ATP2C2, IGHV3-49, CLCN1, CACNG6, PRRG1, FCHO2, BSND, FCGR1A, LINGO1, NLGN4X, IGHV3-53, CD6, CD5, ADGRD2, RAB13, ERVH48-1, NECTIN3, NECTIN2 |
| GO:0062023 | collagen-containing extracellular matrix | 28 | COL17A1, SPON1, PZP, S100A7A, TIMP3, CTSG, DEFA1B, A2M, ELANE, COL28A1, SERPINB1, GDF15, PRG2, DEFA1, PRG3, MMP8, MMP9, HRNR, GH1, PLSCR1, SLPI, COL5A2, SERPING1, SPARCL1, PRTN3, MXRA7, S100A9, S100A8 |
| GO:0042571 | immunoglobulin complex, circulating | 6 | IGHG3, IGHM, IGHG4, IGHG1, IGHG2, IGHA1 |
| GO:0009986 | cell surface | 37 | IGHM, HPN, LRP4, SLC1A3, IL27, LRRC32, LILRA5, LYZL6, PLAU, EPCAM, LAMP3, CLEC5A, ENPP1, CCR7, ITGB7, SCN5A, ELANE, EPHA4, NLGN4X, FCER1G, FCRL5, SCARA5, WNT7A, PSG3, PSG2, RGMA, GRIN1, CEACAM1, CD6, CEACAM6, PSG4, TRPV4, ITGA7, CEACAM8, ROR2, CD24, NECTIN2 |
| GO:0071735 | IgG immunoglobulin complex | 5 | IGHG3, IGHG4, IGHG1, IGHG2, IGHA1 |
| Molecular Function | | | |
| GO:0003823 | antigen binding | 18 | IGHM, IGHV3-73, IGHV3-30, IGHV3-53, IGHV3-33, IGHV4-34, IGHV4-39, IGHV1-24, IGHV2-70D, IGHV3-49, IGHV3-16, IGHG3, IGHG4, IGHG1, IGHG2, IGHA1, IL7R, IGHV3-64D |
| GO:0034987 | immunoglobulin receptor binding | 8 | IGHG3, IGHM, IGHG4, IGHG1, CLEC4D, IGHG2, TARM1, IGHA1 |
| KEGG pathway | | | |
| hsa04060 | Cytokine-cytokine receptor interaction | 25 | IL1RN, AMHR2, CSH2, IL26, CSH1, IL27, CXCL3, CXCL2, TNFSF13B, CCL8, IL36A, CCL2, CCR7, CCL20, GDF15, OSM, INHBA, EDAR, GH1, BMP3, CXCL10, CXCL11, IL5, IL1B, IL7R |
| hsa04657 | IL-17 signaling pathway | 13 | CCL20, CXCL3, CXCL2, MMP9, S100A7A, FOSL1, CXCL10, IL5, IL1B, LCN2, CCL2, S100A9, S100A8 |

Abbreviations: DEGs, differentially expressed genes; GO, gene ontology; KEGG, Kyoto Encyclopedia of Genes and Genomes.
