# Supplementary material for: Identification and validation of diagnostic markers and drugs for pediatric bronchopulmonary dysplasia based on integrating bioinformatics and molecular docking analysis
Source: PLoS One. 2025 May 7;20(5):e0323006. doi: 10.1371/journal.pone.0323006 (PMC12057968; doi:10.1371/journal.pone.0323006)
Supplement: S2 Table — (DOCX) [file pone.0323006.s002.docx]

S2 Table. Complete list of Top108 DEGs from Degree,Closeness and Betweenness algorithms via CytoHubba plug-in.

| Betweenness | BottleNeck | Closeness | ClusteringCeofficient | Dregree | DMNC | EeCentricity | EPC | MCC | MNC | Radiality | Stress |
| --- | --- | --- | --- | --- | --- | --- | --- | --- | --- | --- | --- |
| H3C12 | MPO | IL1B | AR | IL1B | IFI44L | KLKB1 | LCN2 | IFI44L | IL1B | MPO | AR |
| IL1B | H3C12 | MPO | CYP19A1 | CXCL10 | IFI44 | AR | ISG15 | IFI44 | CXCL10 | IL1B | CYP19A1 |
| CCNB1 | IL1B | MMP9 | UGT2A3 | ELANE | IFIT3 | H3C12 | CXCL11 | IFIT3 | ELANE | MMP9 | UGT2A3 |
| MPO | CCNB1 | CXCL10 | ADH6 | CCNB1 | ISG15 | A2M | CXCL3 | ISG15 | MPO | ELANE | ADH6 |
| CXCL10 | ELANE | ELANE | ALDH3B2 | MPO | OASL | CCNB1 | CXCL2 | OASL | DEFA4 | LCN2 | ALDH3B2 |
| AR | CXCL10 | CCL2 | H3C12 | DEFA4 | USP18 | IGHV3-16 | CCR7 | USP18 | CCNB1 | A2M | H3C12 |
| MMP9 | MMP9 | LCN2 | IL1B | DLGAP5 | IFIT2 | SERPING1 | CXCL10 | IFIT2 | DLGAP5 | H3C12 | IL1B |
| ELANE | AR | MMP8 | CXCL10 | MELK | IFIT1 | ELANE | MMP8 | IFIT1 | MELK | MMP8 | CXCL10 |
| RRM2 | LCN2 | A2M | MPO | CCL2 | EPSTI1 | NR3C2 | EPSTI1 | EPSTI1 | CCL2 | RETN | MPO |
| CYP19A1 | IL7R | CTSG | EPHX2 | NCAPG | OAS3 | ETS1 | OAS3 | OAS3 | NCAPG | S100A12 | EPHX2 |
| AK5 | RNASE3 | DEFA4 | MMP9 | MMP9 | HERC5 | CYP19A1 | HERC5 | HERC5 | ISG15 | CXCL10 | MMP9 |
| UGT2A3 | RRM2 | CCR7 | KLKB1 | CTSG | IFI6 | NKX3-1 | USP18 | IFI6 | MMP9 | RNASE3 | KLKB1 |
| CD5 | CYP19A1 | RNASE3 | CCNB1 | ISG15 | ELANE | H2AC14 | IFI6 | ELANE | CTSG | CCL2 | CCNB1 |
| RNASE3 | CD274 | H3C12 | FUT2 | MMP8 | MPO | MORC3 | OASL | MPO | CEACAM8 | CTSG | FUT2 |
| IL7R | UGT2A3 | CAMP | A2M | IFI44L | DEFA4 | H3C8 | IL1B | DEFA4 | LCN2 | CAMP | A2M |
| CCR7 | AK5 | IL5 | ELANE | RRM2 | BPI | MPO | IFI44L | BPI | MMP8 | IL5 | ELANE |
| ENPP1 | CD5 | PRTN3 | CD5 | CEACAM8 | CTSG | CEBPE | IFI44 | CTSG | OASL | PRTN3 | CD5 |
| ADH6 | FCGR1A | RETN | RRM2 | LCN2 | AZU1 | S100A12 | ELANE | AZU1 | IFI44L | CCR7 | RRM2 |
| ALDH3B2 | IL5 | CXCL11 | AK5 | OASL | CXCL10 | LCN2 | IFIT3 | CXCL10 | IFI44 | DEFA4 | AK5 |
| CLEC4E | PRG2 | CEACAM8 | CCR7 | IFI44 | CEACAM8 | RETN | MMP9 | CEACAM8 | IFIT3 | HP | CCR7 |
| KLKB1 | ENPP1 | S100A12 | ENPP1 | IFIT3 | MS4A3 | SERPINB1 | IFIT2 | MS4A3 | KIF15 | CEACAM8 | ENPP1 |
| CD274 | ADH6 | BPI | IGHV3-16 | KIF15 | RNASE3 | SLPI | IFIT1 | RNASE3 | CAMP | BPI | IGHV3-16 |
| IL5 | CLEC4E | CCNB1 | GAD1 | CAMP | DLGAP5 | MMP8 | CCL2 | DLGAP5 | USP18 | CD274 | GAD1 |
| PRG2 | PAPPA | AZU1 | ISG15 | USP18 | MELK | NCAPH | AZU1 | MELK | DEPDC1 | AZU1 | ISG15 |
| CCL2 | ALDH3B2 | CD274 | CD24 | DEPDC1 | NCAPG | DLGAP5 | CTSG | NCAPG | SPC25 | CXCL11 | CD24 |
| PAPPA | IGF2 | CXCL2 | CCL2 | SPC25 | CCNB1 | NCAPG | MPO | CCNB1 | IFIT2 | MS4A3 | CCL2 |
| IGHV3-16 | CCL2 | CCL20 | IL7R | IFIT2 | KIF15 | GINS1 | CAMP | KIF15 | IFIT1 | IL7R | IL7R |
| A2M | KLKB1 | MS4A3 | IFI44L | IFIT1 | NUSAP1 | CCNE2 | MS4A3 | NUSAP1 | NUSAP1 | CXCL2 | IFI44L |
| HP | IFI44L | IL7R | EPCAM | NUSAP1 | DEPDC1 | IL1B | CEACAM8 | DEPDC1 | RRM2 | CCL20 | EPCAM |
| ARG1 | IGHV3-16 | CXCL3 | OASL | RNASE3 | KIF23 | KNL1 | DEFA4 | KIF23 | RNASE3 | IDO1 | OASL |
| GBP5 | HP | HP | GBP5 | BPI | CAMP | PCLAF | RNASE3 | CAMP | BPI | IGHV3-16 | GBP5 |
| LCN2 | ARG1 | IDO1 | RNASE3 | PRTN3 | CDKN3 | DEPDC1 | BPI | CDKN3 | CXCL11 | CXCL3 | RNASE3 |
| MMP8 | ITK | ISG15 | IFI44 | CXCL11 | PRTN3 | PLAU | PRTN3 | PRTN3 | CCR7 | PLAU | IFI44 |
| DEFA4 | GBP5 | ARG1 | IFIT3 | CCR7 | NCAPH | IL27 | A2M | NCAPH | EPSTI1 | SERPING1 | IFIT3 |
| FCGR1A | S100A12 | PLAU | CCL8 | EPSTI1 | SPC25 | SPC25 | CCL20 | SPC25 | OAS3 | ARG1 | CCL8 |
| FCER1G | TNNI3 | IGHV3-16 | PRG2 | OAS3 | RRM2 | ROR2 | CCL8 | RRM2 | HERC5 | SLPI | PRG2 |
| ITK | EPHX2 | IFI44L | PAPPA | HERC5 | CCL2 | MMP9 | RETN | CCL2 | IFI6 | CLEC4E | PAPPA |
| ATF3 | BATF2 | CLEC4E | BATF2 | IFI6 | CCR7 | SKA3 | PGLYRP1 | CCR7 | PRTN3 | KLKB1 | BATF2 |
| BATF2 | BRIP1 | OASL | USP18 | KIF23 | CXCL11 | HP | IL5 | CXCL11 | DTL | CEBPE | USP18 |
| TNNI3 | CD24 | IFI44 | IFIT2 | AZU1 | CXCL2 | KIF15 | IDO1 | CXCL2 | PCLAF | OSM | IFIT2 |
| EPHX2 | FCER1G | IFIT3 | IFIT1 | DTL | CCL20 | KIF23 | IL7R | CCL20 | KIF23 | ATF3 | IFIT1 |
| CD24 | CHIT1 | ATF3 | HPRT1 | PCLAF | IL1B | NUSAP1 | OLFM4 | IL1B | CDKN3 | TNFAIP6 | HPRT1 |
| CHIT1 | ATF3 | USP18 | GYG1 | CDKN3 | CXCL3 | UGT2A3 | S100A12 | CXCL3 | AZU1 | IL1RN | GYG1 |
| EPX | CEACAM8 | IFIT2 | CD79A | MS4A3 | PCLAF | CDKN3 | CD274 | PCLAF | MS4A3 | CCNB1 | CD79A |
| CTSG | DEFA4 | IFIT1 | CD274 | NCAPH | DTL | MELK | RNASE2 | DTL | NCAPH | LY96 | CD274 |
| CEACAM8 | DEFA1 | TNFAIP6 | CXCL11 | SKA3 | SKA3 | CDC25C | DEFA1 | SKA3 | SKA3 | AIM2 | CXCL11 |
| IGF2 | UGCG | CCL8 | TNNI3 | CXCL2 | MND1 | RRM2 | EPX | MND1 | CXCL2 | EPX | TNNI3 |
| S100A12 | RETN | OSM | CLEC4E | CCL20 | MMP8 | HSD17B2 | ARG1 | MMP8 | CCL20 | TIMP3 | CLEC4E |
| ISG15 | ISG15 | SLPI | FCGR1A | A2M | CCL8 | AZU1 | HP | CCL8 | A2M | AR | FCGR1A |
| PRTN3 | CXCL11 | LY96 | ITK | MND1 | MMP9 | CTSG | PRG2 | MMP9 | MND1 | DEFA1 | ITK |
| KIF23 | CSH2 | SERPING1 | IGF2 | CXCL3 | CDC25C | CAMP | TNFAIP6 | CDC25C | CXCL3 | OLFM4 | IGF2 |
| AZU1 | CXCL2 | IL1RN | ARG1 | IL5 | LCN2 | MS4A3 | PLAU | LCN2 | CCL8 | SERPINB1 | ARG1 |
| TIMP3 | HPRT1 | EPX | LCN2 | CCL8 | PGLYRP1 | CEACAM8 | PLSCR1 | PGLYRP1 | RETN | PGLYRP1 | LCN2 |
| HPRT1 | GYG1 | AIM2 | CHIT1 | HP | KNL1 | DEFA4 | H3C12 | KNL1 | CSH1 | TCN1 | CHIT1 |
| GYG1 | MMP8 | PGLYRP1 | FCER1G | IL7R | CSH1 | RNASE3 | SLPI | CSH1 | CSH2 | CCL8 | FCER1G |
| UGCG | TIMP3 | OLFM4 | IL5 | H3C12 | CSH2 | BPI | DEFA1B | CSH2 | KNL1 | ISG15 | IL5 |
| GAD1 | NCAPG | CEBPE | MELK | FCER1G | PSG2 | PRTN3 | SERPINB1 | PSG2 | CDC25C | MORC3 | MELK |
| EPCAM | GINS1 | TIMP3 | RETN | S100A12 | PSG4 | DEFA1 | KLKB1 | PSG4 | IL7R | H3C8 | RETN |
| CD79A | EPX | DLGAP5 | MMP8 | CD5 | PSG3 | KIF21A | SERPING1 | PSG3 | IDO1 | H2AC14 | MMP8 |
| PLCG1 | DEPDC1 | MELK | PLCG1 | AR | RETN | MND1 | DEFA3 | RETN | PGLYRP1 | PRG2 | PLCG1 |
| FUT2 | IFIT3 | KLKB1 | UGCG | CD274 | A2M | DTL | CEACAM6 | A2M | DEFA1 | OASL | UGCG |
| MELK | KIF23 | DEFA1 | DEFA4 | RETN | IDO1 | POLE2 | S100A8 | IDO1 | S100A12 | IFI44L | DEFA4 |
| IFI44L | IFIT1 | RRM2 | DLGAP5 | CSH1 | S100A12 | CLEC4E | S100A9 | S100A12 | PSG2 | IFI44 | DLGAP5 |
| CCL8 | NUSAP1 | AR | NCAPG | CSH2 | CD274 | TCN1 | LY96 | CD274 | PSG4 | IFIT3 | NCAPG |
| IFI44 | GAD1 | NCAPG | PCLAF | EPX | OLFM4 | TNFAIP6 | CD5 | OLFM4 | PSG3 | USP18 | PCLAF |
| IFIT3 | EPCAM | CD5 | KIF15 | KNL1 | DEFA1 | IL1RN | SIGLEC1 | DEFA1 | IL5 | IFIT2 | KIF15 |
| PCLAF | CD79A | SERPINB1 | SPC25 | ENPP1 | IL5 | CXCL11 | IL1RN | IL5 | EPX | IFIT1 | SPC25 |
| CXCL11 | AZU1 | NUSAP1 | CXCL2 | CDC25C | IL7R | CXCL3 | UBD | IL7R | RNASE2 | RNASE2 | CXCL2 |
| OASL | CTSG | KIF15 | CCL20 | ARG1 | FCER1G | IL5 | CEBPE | FCER1G | HP | CD5 | CCL20 |
| CAMP | CAMP | UBD | CEACAM8 | IDO1 | EPX | S100A8 | ATF3 | EPX | ARG1 | S100A8 | CEACAM8 |
| DEPDC1 | PLCG1 | GBP5 | EPX | PGLYRP1 | HP | CXCL2 | TCN1 | HP | CD274 | S100A9 | EPX |
| GINS1 | PRTN3 | TCN1 | TIMP3 | DEFA1 | ARG1 | S100A9 | FCGR1A | ARG1 | OLFM4 | GBP5 | TIMP3 |
| DLGAP5 | FUT2 | KIF23 | ATF3 | CLEC4E | RNASE2 | CCR7 | OSM | RNASE2 | PLSCR1 | UBD | ATF3 |
| LY96 | KIF21A | DEPDC1 | CTSG | PSG2 | CLEC4E | CXCL10 | CEACAM1 | CLEC4E | CLEC4D | SERPINB10 | CTSG |
| MS4A3 | MND1 | SPC25 | KIF23 | PSG4 | PRG2 | CCL20 | IGHV3-16 | PRG2 | CLEC4E | FCGR1A | KIF23 |
| USP18 | DTL | PRG2 | NUSAP1 | PSG3 | H3C12 | SERPINB10 | TIMP3 | H3C12 | CLEC6A | IL26 | NUSAP1 |
| IFIT2 | CEBPE | PCLAF | GINS1 | KLKB1 | PLSCR1 | CUBN | AR | PLSCR1 | TNFAIP6 | CD177 | GINS1 |
| IFIT1 | PLSCR1 | CDKN3 | DEPDC1 | PRG2 | CLEC4D | TIMP3 | CLEC4E | CLEC4D | S100A8 | CEACAM6 | DEPDC1 |
| NCAPG | FANCL | FCGR1A | SKA3 | IGHV3-16 | CLEC6A | EPX | FCER1G | CLEC6A | S100A9 | DEFA3 | SKA3 |
| SPC25 | BSND | NCAPH | HP | RNASE2 | TNFAIP6 | PRG2 | APOC1 | TNFAIP6 | SERPINB1 | DEFA1B | HP |
| KIF15 | CHMP4C | RNASE2 | PRTN3 | FCGR1A | SERPINB1 | RNASE2 | CLEC6A | SERPINB1 | SLPI | APOC1 | PRTN3 |
| DEFA1 | CLCN1 | EPSTI1 | AZU1 | ATF3 | PLAU | OSM | HPR | PLAU | PRG2 | HPR | AZU1 |
| RETN | VPS37D | OAS3 | CAMP | OLFM4 | CD5 | ADH6 | CLEC4D | CD5 | SERPING1 | CUBN | CAMP |
| BPI | CHRNA9 | HERC5 | S100A12 | PLSCR1 | AR | AIM2 | ITK | AR | PLAU | CEACAM1 | S100A12 |
| SKA3 | CHRNB3 | IFI6 | BPI | POLE2 | DEFA3 | LY96 | PAPPA | DEFA3 | FCER1G | MTUS1 | BPI |
| NUSAP1 | POLE2 | SKA3 | MS4A3 | CLEC4D | DEFA1B | AK5 | MORC3 | DEFA1B | DEFA3 | ITK | MS4A3 |
| DTL | CLDN1 | FCER1G | DEFA1 | CLEC6A | ENPP1 | IL7R | H3C8 | ENPP1 | DEFA1B | FCER1G | DEFA1 |
| IDO1 | TACSTD2 | MORC3 | LY96 | TNFAIP6 | S100A8 | APOC1 | IGF2 | S100A8 | CEACAM6 | SH3GL2 | LY96 |
| CXCL2 | CLEC4D | H3C8 | IDO1 | S100A8 | S100A9 | CD177 | FCAR | S100A9 | CEBPE | RRM2 | IDO1 |
| CCL20 | KRT72 | CEACAM6 | DTL | TNNI3 | SLPI | HPR | DTL | SLPI | POLE2 | PRDM1 | DTL |
| SERPING1 | KRT73 | S100A8 | CDKN3 | S100A9 | KLKB1 | GLRX | CCNB1 | KLKB1 | TCN1 | DLGAP5 | CDKN3 |
| CD6 | CLEC6A | S100A9 | RNASE2 | SERPINB1 | IGHV3-16 | ARG1 | NCAPH | IGHV3-16 | GH1 | MELK | RNASE2 |
| CDKN3 | SPTLC3 | DEFA3 | SERPING1 | SLPI | SERPING1 | CCL2 | DLGAP5 | SERPING1 | IL1RN | IL27 | SERPING1 |
| RNASE2 | EIF1AY | DEFA1B | CD6 | TIMP3 | FCGR1A | CD274 | NCAPG | FCGR1A | TNNI3 | ROR2 | CD6 |
| NCAPH | ZFY | H2AC14 | OLFM4 | GINS1 | ATF3 | DEFA3 | PCLAF | ATF3 | GINS1 | NCAPG | OLFM4 |
| CEACAM6 | CLEC5A | SERPINB10 | NCAPH | PAPPA | CEACAM6 | DEFA1B | DEPDC1 | CEACAM6 | CCNE2 | CLEC4D | NCAPH |
| CSH1 | TCN1 | IL26 | CEACAM6 | SERPING1 | POLE2 | IDO1 | SPC25 | POLE2 | UBD | CLEC6A | CEACAM6 |
| CSH2 | TNFAIP6 | KNL1 | SIGLEC1 | PLAU | TNNI3 | ATF3 | KIF15 | TNNI3 | KLKB1 | NUSAP1 | SIGLEC1 |
| SIGLEC1 | CSH1 | CDC25C | SLPI | ALDH3B2 | TIMP3 | SH3GL2 | KIF23 | TIMP3 | OSM | KIF23 | SLPI |
| BRIP1 | GH1 | ITK | PGLYRP1 | LY96 | GINS1 | CEACAM1 | NUSAP1 | GINS1 | FCAR | CHIT1 | PGLYRP1 |
| POLE2 | IL1RN | GINS1 | KNL1 | IGF2 | PAPPA | CEACAM6 | CDKN3 | PAPPA | LY96 | PCLAF | KNL1 |
| OLFM4 | PSG2 | CD177 | MND1 | BATF2 | ALDH3B2 | NEIL3 | MELK | ALDH3B2 | CD5 | NCAPH | MND1 |
| KNL1 | PSG4 | CEACAM1 | POLE2 | CYP19A1 | LY96 | OLFM4 | RRM2 | LY96 | RBM20 | CDKN3 | POLE2 |
| SLPI | PSG3 | CCNE2 | CSH1 | DEFA3 | IGF2 | PGLYRP1 | MND1 | IGF2 | SCN5A | KIF15 | CSH1 |
| S100A8 | LRP4 | CLEC4D | CSH2 | DEFA1B | BATF2 | CLEC4D | SKA3 | BATF2 | APOC1 | DEPDC1 | CSH2 |
| S100A9 | CXCL3 | CLEC6A | S100A8 | ITK | CYP19A1 | CLEC6A | CDC25C | CYP19A1 | HPR | SPC25 | S100A8 |
| PGLYRP1 | WNT7A | MTUS1 | S100A9 | CEACAM6 | ITK | ISG15 | KNL1 | ITK | H3C12 | GINS1 | S100A9 |
| MND1 | S100A8 | DTL | BRIP1 | CEBPE | CEBPE | CCL8 | CD6 | CEBPE | FCGR1A | CCNE2 | BRIP1 |

Abbreviations: DEGs, differentially expressed genes.
