# Supplementary material for: Identification and validation of diagnostic markers and drugs for pediatric bronchopulmonary dysplasia based on integrating bioinformatics and molecular docking analysis
Source: PLoS One. 2025 May 7;20(5):e0323006. doi: 10.1371/journal.pone.0323006 (PMC12057968; doi:10.1371/journal.pone.0323006)
Supplement: S3 Table — (DOCX) [file pone.0323006.s003.docx]

S3 Table. Complete list of three machine learning algorithms

| Lasso | SVM-RFE | RF | intersection |
| --- | --- | --- | --- |
| CCNB1 | IL7R | IL7R | CCNB1 |
| CXCL10 | MPO | KLKB1 | CXCL10 |
| IL7R | CXCL10 | CCNB1 | IL7R |
| KLKB1 | CD274 | IL5 | DEFA4 |
| DEFA4 | NCAPG | NCAPG | PRTN3 |
| PRTN3 | CCNB1 | DEFA4 | NCAPG |
| NCAPG | ISG15 | KIF23 |  |
| NUSAP1 | PRTN3 | DEFA1 |  |
| S100A8 | DEFA1 | CEACAM8 |  |
|  | AZU1 | NUSAP1 |  |
|  | DEFA4 | S100A8 |  |
|  | LCN2 | CXCL10 |  |
|  | RRM2 | PRTN3 |  |
|  | PRG2 | CAMP |  |
|  | H3C12 | DEPDC1 |  |
|  | ELANE |  |  |
|  | CEACAM8 |  |  |
|  | IL1B |  |  |
|  | S100A12 |  |  |
|  | HP |  |  |
|  | CAMP |  |  |
|  | CCL2 |  |  |

Abbreviations: SVM-RFE,Support Vector Machine-Recursive Feature Elimination; LASSO,Least Absolute Shrinkage and Selection Operator; RF,Random Forest.
