# Supplementary material for: Identification and validation of diagnostic markers and drugs for pediatric bronchopulmonary dysplasia based on integrating bioinformatics and molecular docking analysis
Source: PLoS One. 2025 May 7;20(5):e0323006. doi: 10.1371/journal.pone.0323006 (PMC12057968; doi:10.1371/journal.pone.0323006)
Supplement: S4 Table — (DOCX) [file pone.0323006.s004.docx]

S4 Table. Complete list of ssGSEA results (adj.P value <0.05).

| Hallmark ID | NES |
| --- | --- |
| **ssGSEA based on IL7R** | |
| HALLMARK_MYC_TARGETS_V1 | 1.444813489 |
| HALLMARK_OXIDATIVE_PHOSPHORYLATION | 1.360008912 |
| HALLMARK_DNA_REPAIR | 1.34404146 |
| HALLMARK_ALLOGRAFT_REJECTION | 1.280154185 |
| HALLMARK_UNFOLDED_PROTEIN_RESPONSE | 1.350287589 |
| HALLMARK_MTORC1_SIGNALING | 1.273773887 |
| HALLMARK_FATTY_ACID_METABOLISM | 1.288523295 |
| HALLMARK_PI3K_AKT_MTOR_SIGNALING | 1.349058237 |
| HALLMARK_INTERFERON_GAMMA_RESPONSE | 1.215499389 |
| HALLMARK_ADIPOGENESIS | 1.206143276 |
| HALLMARK_E2F_TARGETS | 1.193150522 |
| HALLMARK_PROTEIN_SECRETION | 1.28168804 |
| HALLMARK_MITOTIC_SPINDLE | 1.179066538 |
| HALLMARK_P53_PATHWAY | 1.177953148 |
| HALLMARK_MYC_TARGETS_V2 | 1.335295241 |
| HALLMARK_IL2_STAT5_SIGNALING | 1.165404753 |
| HALLMARK_ANDROGEN_RESPONSE | 1.205670018 |
| HALLMARK_INTERFERON_ALPHA_RESPONSE | 1.189016526 |
| HALLMARK_UV_RESPONSE_UP | 1.147303572 |
| HALLMARK_APOPTOSIS | 1.145595151 |
| HALLMARK_PEROXISOME | 1.154394678 |
| HALLMARK_UV_RESPONSE_DN | 1.134323214 |
| HALLMARK_CHOLESTEROL_HOMEOSTASIS | 1.174183183 |
| HALLMARK_G2M_CHECKPOINT | 1.10858482 |
| HALLMARK_INFLAMMATORY_RESPONSE | 1.096227682 |
| HALLMARK_GLYCOLYSIS | 1.096040386 |
| HALLMARK_APICAL_JUNCTION | 1.092542759 |
| HALLMARK_COMPLEMENT | 1.091415255 |
| HALLMARK_IL6_JAK_STAT3_SIGNALING | 1.124084381 |
| **ssGSEA based on DEFA4** | |
| HALLMARK_OXIDATIVE_PHOSPHORYLATION | 2.075169198 |
| HALLMARK_MTORC1_SIGNALING | 2.053083413 |
| HALLMARK_E2F_TARGETS | 2.017637338 |
| HALLMARK_MYC_TARGETS_V1 | 1.996328327 |
| HALLMARK_CHOLESTEROL_HOMEOSTASIS | 1.985446852 |
| HALLMARK_G2M_CHECKPOINT | 1.982756724 |
| HALLMARK_PROTEIN_SECRETION | 1.967172376 |
| HALLMARK_IL6_JAK_STAT3_SIGNALING | 1.958249872 |
| HALLMARK_DNA_REPAIR | 1.897696729 |
| HALLMARK_ADIPOGENESIS | 1.893684187 |
| HALLMARK_PI3K_AKT_MTOR_SIGNALING | 1.886430487 |
| HALLMARK_COMPLEMENT | 1.885999843 |
| HALLMARK_TNFA_SIGNALING_VIA_NFKB | 1.882268751 |
| HALLMARK_INFLAMMATORY_RESPONSE | 1.863623624 |
| HALLMARK_MITOTIC_SPINDLE | 1.826566219 |
| HALLMARK_APOPTOSIS | 1.813497694 |
| HALLMARK_UNFOLDED_PROTEIN_RESPONSE | 1.785417021 |
| HALLMARK_UV_RESPONSE_UP | 1.779859679 |
| HALLMARK_GLYCOLYSIS | 1.772562886 |
| HALLMARK_FATTY_ACID_METABOLISM | 1.771402746 |
| HALLMARK_P53_PATHWAY | 1.744952285 |
| HALLMARK_HYPOXIA | 1.694472554 |
| HALLMARK_XENOBIOTIC_METABOLISM | 1.611165219 |
| HALLMARK_ANDROGEN_RESPONSE | 1.797740934 |
| HALLMARK_INTERFERON_GAMMA_RESPONSE | 1.598957379 |
| HALLMARK_IL2_STAT5_SIGNALING | 1.585268547 |
| HALLMARK_REACTIVE_OXYGEN_SPECIES_PATHWAY | 1.932419784 |
| HALLMARK_PEROXISOME | 1.718544771 |
| HALLMARK_ALLOGRAFT_REJECTION | 1.539439255 |
| HALLMARK_ESTROGEN_RESPONSE_LATE | 1.527782037 |
| HALLMARK_COAGULATION | 1.59570221 |
| HALLMARK_APICAL_JUNCTION | 1.461630412 |
| HALLMARK_KRAS_SIGNALING_UP | 1.472932349 |
| HALLMARK_ANGIOGENESIS | 1.774099152 |
| HALLMARK_INTERFERON_ALPHA_RESPONSE | 1.553793064 |
| HALLMARK_ESTROGEN_RESPONSE_EARLY | 1.410343896 |
| HALLMARK_EPITHELIAL_MESENCHYMAL_TRANSITION | 1.414477616 |
| HALLMARK_HEME_METABOLISM | 1.403282932 |
| HALLMARK_MYC_TARGETS_V2 | 1.597845093 |
| HALLMARK_BILE_ACID_METABOLISM | 1.461817279 |
| HALLMARK_UV_RESPONSE_DN | 1.41318532 |
| HALLMARK_TGF_BETA_SIGNALING | 1.53571657 |
| HALLMARK_NOTCH_SIGNALING | 1.561150844 |
| HALLMARK_SPERMATOGENESIS | 1.294757291 |
| HALLMARK_APICAL_SURFACE | 1.466846542 |
| HALLMARK_MYOGENESIS | 1.228044991 |
| **ssGSEA based on CXCL10** | |
| HALLMARK_INTERFERON_ALPHA_RESPONSE | 2.753786813 |
| HALLMARK_INTERFERON_GAMMA_RESPONSE | 2.699364012 |
| HALLMARK_TNFA_SIGNALING_VIA_NFKB | 2.184539217 |
| HALLMARK_INFLAMMATORY_RESPONSE | 2.143243047 |
| HALLMARK_IL6_JAK_STAT3_SIGNALING | 2.062455795 |
| HALLMARK_ALLOGRAFT_REJECTION | 1.994083064 |
| HALLMARK_E2F_TARGETS | 1.960054714 |
| HALLMARK_G2M_CHECKPOINT | 1.88595007 |
| HALLMARK_COMPLEMENT | 1.884777401 |
| HALLMARK_APOPTOSIS | 1.876701901 |
| HALLMARK_MTORC1_SIGNALING | 1.710922817 |
| HALLMARK_MYC_TARGETS_V1 | 1.700713953 |
| HALLMARK_OXIDATIVE_PHOSPHORYLATION | 1.647002429 |
| HALLMARK_IL2_STAT5_SIGNALING | 1.637409042 |
| HALLMARK_DNA_REPAIR | 1.670836608 |
| HALLMARK_CHOLESTEROL_HOMEOSTASIS | 1.778579777 |
| HALLMARK_P53_PATHWAY | 1.548696531 |
| HALLMARK_MITOTIC_SPINDLE | 1.528256825 |
| HALLMARK_KRAS_SIGNALING_UP | 1.514435225 |
| HALLMARK_UNFOLDED_PROTEIN_RESPONSE | 1.539234722 |
| HALLMARK_ANDROGEN_RESPONSE | 1.571103658 |
| HALLMARK_UV_RESPONSE_UP | 1.46067191 |
| HALLMARK_FATTY_ACID_METABOLISM | 1.435867738 |
| HALLMARK_ESTROGEN_RESPONSE_LATE | 1.392108513 |
| HALLMARK_PROTEIN_SECRETION | 1.504888744 |
| HALLMARK_PI3K_AKT_MTOR_SIGNALING | 1.452160076 |
| HALLMARK_REACTIVE_OXYGEN_SPECIES_PATHWAY | 1.543264378 |
| HALLMARK_SPERMATOGENESIS | 1.368876155 |
| HALLMARK_GLYCOLYSIS | 1.284480237 |
| **ssGSEA based on CCNB1** | |
| HALLMARK_MYC_TARGETS_V1 | 2.077777277 |
| HALLMARK_E2F_TARGETS | 2.014904676 |
| HALLMARK_OXIDATIVE_PHOSPHORYLATION | 2.010408008 |
| HALLMARK_G2M_CHECKPOINT | 1.916536471 |
| HALLMARK_PROTEIN_SECRETION | 1.89728436 |
| HALLMARK_MTORC1_SIGNALING | 1.856903673 |
| HALLMARK_FATTY_ACID_METABOLISM | 1.725864793 |
| HALLMARK_DNA_REPAIR | 1.718425595 |
| HALLMARK_ANDROGEN_RESPONSE | 1.705120104 |
| HALLMARK_UNFOLDED_PROTEIN_RESPONSE | 1.696599142 |
| HALLMARK_ADIPOGENESIS | 1.582607454 |
| HALLMARK_MITOTIC_SPINDLE | 1.550256506 |
| HALLMARK_GLYCOLYSIS | 1.531420826 |
| HALLMARK_UV_RESPONSE_UP | 1.539523835 |
| HALLMARK_P53_PATHWAY | 1.472216628 |
| HALLMARK_PI3K_AKT_MTOR_SIGNALING | 1.610759807 |
| HALLMARK_HEME_METABOLISM | 1.464892572 |
| HALLMARK_INTERFERON_GAMMA_RESPONSE | 1.452249904 |
| HALLMARK_PEROXISOME | 1.584153763 |
| HALLMARK_ALLOGRAFT_REJECTION | 1.432031859 |
| HALLMARK_APOPTOSIS | 1.429711907 |
| HALLMARK_TNFA_SIGNALING_VIA_NFKB | 1.382929798 |
| HALLMARK_IL2_STAT5_SIGNALING | 1.383738601 |
| HALLMARK_COMPLEMENT | 1.367528313 |
| HALLMARK_MYC_TARGETS_V2 | 1.571395499 |
| HALLMARK_XENOBIOTIC_METABOLISM | 1.350124391 |
| HALLMARK_HYPOXIA | 1.340385898 |
| HALLMARK_INFLAMMATORY_RESPONSE | 1.337598927 |
| HALLMARK_BILE_ACID_METABOLISM | 1.401087222 |
| HALLMARK_CHOLESTEROL_HOMEOSTASIS | 1.500893659 |
| HALLMARK_ESTROGEN_RESPONSE_LATE | 1.329864956 |
| HALLMARK_KRAS_SIGNALING_UP | 1.302344035 |
| HALLMARK_UV_RESPONSE_DN | 1.352753515 |
| HALLMARK_INTERFERON_ALPHA_RESPONSE | 1.39571015 |
| HALLMARK_REACTIVE_OXYGEN_SPECIES_PATHWAY | 1.51120713 |
| HALLMARK_SPERMATOGENESIS | 1.339935554 |
| HALLMARK_TGF_BETA_SIGNALING | 1.418569884 |
| HALLMARK_IL6_JAK_STAT3_SIGNALING | 1.320193218 |
| **ssGSEA based on PRTN3** | |
| HALLMARK_CHOLESTEROL_HOMEOSTASIS | 1.932819389 |
| HALLMARK_MTORC1_SIGNALING | 1.919250944 |
| HALLMARK_TNFA_SIGNALING_VIA_NFKB | 1.888251816 |
| HALLMARK_E2F_TARGETS | 1.841790754 |
| HALLMARK_G2M_CHECKPOINT | 1.829763641 |
| HALLMARK_INFLAMMATORY_RESPONSE | 1.824396372 |
| HALLMARK_ADIPOGENESIS | 1.823593535 |
| HALLMARK_OXIDATIVE_PHOSPHORYLATION | 1.818724736 |
| HALLMARK_IL6_JAK_STAT3_SIGNALING | 1.814762458 |
| HALLMARK_COMPLEMENT | 1.80532802 |
| HALLMARK_PROTEIN_SECRETION | 1.787644501 |
| HALLMARK_PI3K_AKT_MTOR_SIGNALING | 1.783570588 |
| HALLMARK_MITOTIC_SPINDLE | 1.776793586 |
| HALLMARK_DNA_REPAIR | 1.710146688 |
| HALLMARK_APOPTOSIS | 1.699057178 |
| HALLMARK_UV_RESPONSE_UP | 1.694392552 |
| HALLMARK_GLYCOLYSIS | 1.689593217 |
| HALLMARK_HYPOXIA | 1.663405901 |
| HALLMARK_MYC_TARGETS_V1 | 1.656517238 |
| HALLMARK_P53_PATHWAY | 1.633483942 |
| HALLMARK_FATTY_ACID_METABOLISM | 1.630785325 |
| HALLMARK_XENOBIOTIC_METABOLISM | 1.595111781 |
| HALLMARK_ALLOGRAFT_REJECTION | 1.577823698 |
| HALLMARK_HEME_METABOLISM | 1.570502877 |
| HALLMARK_IL2_STAT5_SIGNALING | 1.592395558 |
| HALLMARK_INTERFERON_GAMMA_RESPONSE | 1.564655021 |
| HALLMARK_REACTIVE_OXYGEN_SPECIES_PATHWAY | 1.913101465 |
| HALLMARK_COAGULATION | 1.624009284 |
| HALLMARK_UNFOLDED_PROTEIN_RESPONSE | 1.643061377 |
| HALLMARK_ANDROGEN_RESPONSE | 1.664594876 |
| HALLMARK_ESTROGEN_RESPONSE_LATE | 1.522488187 |
| HALLMARK_PEROXISOME | 1.646284386 |
| HALLMARK_APICAL_JUNCTION | 1.492416924 |
| HALLMARK_ESTROGEN_RESPONSE_EARLY | 1.475822587 |
| HALLMARK_KRAS_SIGNALING_UP | 1.418632952 |
| HALLMARK_EPITHELIAL_MESENCHYMAL_TRANSITION | 1.398303229 |
| HALLMARK_INTERFERON_ALPHA_RESPONSE | 1.521451369 |
| HALLMARK_TGF_BETA_SIGNALING | 1.620517003 |
| HALLMARK_BILE_ACID_METABOLISM | 1.412197615 |
| HALLMARK_UV_RESPONSE_DN | 1.394048058 |
| HALLMARK_ANGIOGENESIS | 1.69495662 |
| HALLMARK_MYOGENESIS | 1.330317493 |
| HALLMARK_NOTCH_SIGNALING | 1.530327112 |
| HALLMARK_MYC_TARGETS_V2 | 1.415434557 |
| HALLMARK_APICAL_SURFACE | 1.452314031 |
| HALLMARK_SPERMATOGENESIS | 1.253859084 |
| HALLMARK_WNT_BETA_CATENIN_SIGNALING | 1.364662568 |
| **ssGSEA based on NCAPG** | |
| HALLMARK_E2F_TARGETS | 4.320174555 |
| HALLMARK_G2M_CHECKPOINT | 4.027997931 |
| HALLMARK_MYC_TARGETS_V1 | 4.025711312 |
| HALLMARK_OXIDATIVE_PHOSPHORYLATION | 3.790167568 |
| HALLMARK_PROTEIN_SECRETION | 3.623200749 |
| HALLMARK_MTORC1_SIGNALING | 3.536322491 |
| HALLMARK_MITOTIC_SPINDLE | 3.267511688 |
| HALLMARK_ANDROGEN_RESPONSE | 3.240441276 |
| HALLMARK_INTERFERON_ALPHA_RESPONSE | 2.86474653 |
| HALLMARK_DNA_REPAIR | 2.816734261 |
| HALLMARK_UNFOLDED_PROTEIN_RESPONSE | 2.792437618 |
| HALLMARK_FATTY_ACID_METABOLISM | 2.668553255 |
| HALLMARK_ADIPOGENESIS | 2.555380974 |
| HALLMARK_INTERFERON_GAMMA_RESPONSE | 2.495657373 |
| HALLMARK_UV_RESPONSE_DN | 2.579015501 |
| HALLMARK_HEME_METABOLISM | 2.371778174 |
| HALLMARK_GLYCOLYSIS | 2.325133023 |
| HALLMARK_IL2_STAT5_SIGNALING | 2.278583463 |
| HALLMARK_PEROXISOME | 2.372170213 |
| HALLMARK_APOPTOSIS | 2.250521272 |
| HALLMARK_PI3K_AKT_MTOR_SIGNALING | 2.350823003 |
| HALLMARK_TNFA_SIGNALING_VIA_NFKB | 2.051670318 |
| HALLMARK_HYPOXIA | 2.044666761 |
| HALLMARK_COMPLEMENT | 1.984566708 |
| HALLMARK_KRAS_SIGNALING_UP | 1.98424623 |
| HALLMARK_UV_RESPONSE_UP | 2.078345825 |
| HALLMARK_P53_PATHWAY | 1.944372676 |
| HALLMARK_ESTROGEN_RESPONSE_LATE | 1.935401782 |
| HALLMARK_CHOLESTEROL_HOMEOSTASIS | 2.216940777 |
| HALLMARK_TGF_BETA_SIGNALING | 2.279120818 |
| HALLMARK_ALLOGRAFT_REJECTION | 1.890278694 |
| HALLMARK_MYC_TARGETS_V2 | 2.111663302 |
| HALLMARK_EPITHELIAL_MESENCHYMAL_TRANSITION | 1.817017497 |
| HALLMARK_SPERMATOGENESIS | 1.856875815 |
| HALLMARK_BILE_ACID_METABOLISM | 1.86221767 |
| HALLMARK_INFLAMMATORY_RESPONSE | 1.68749976 |
| HALLMARK_XENOBIOTIC_METABOLISM | 1.605971348 |
| HALLMARK_REACTIVE_OXYGEN_SPECIES_PATHWAY | 1.753651497 |
