# Supplementary material for: Identification and validation of diagnostic markers and drugs for pediatric bronchopulmonary dysplasia based on integrating bioinformatics and molecular docking analysis
Source: PLoS One. 2025 May 7;20(5):e0323006. doi: 10.1371/journal.pone.0323006 (PMC12057968; doi:10.1371/journal.pone.0323006)
Supplement: S5 Table — (DOCX) [file pone.0323006.s005.docx]

S5 Table. Candidate drug predicted using DSigDB (adj.P value <0.05).

| Drug names | P-value | Adjusted P-value | Genes |
| --- | --- | --- | --- |
| LUCANTHONE CTD 00006227 | 2.33E-05 | 0.008398257 | CXCL10;CCNB1;NCAPG |
| etoposide MCF7 DOWN | 8.41E-05 | 0.010547141 | CCNB1;NCAPG |
| etoposide HL60 DOWN | 8.76E-05 | 0.010547141 | CCNB1;PRTN3 |
| ciclopirox MCF7 DOWN | 1.64E-04 | 0.012071893 | CCNB1;NCAPG |
| Sodium dodecyl sulfate CTD 00006753 | 1.90E-04 | 0.012071893 | CXCL10;IL7R |
| ns-398 CTD 00002958 | 2.01E-04 | 0.012071893 | CXCL10;CCNB1 |
| resveratrol CTD 00002483 | 5.38E-04 | 0.027744012 | CXCL10;CCNB1;NCAPG;IL7R |
| troglitazone CTD 00002415 | 6.38E-04 | 0.028783693 | CXCL10;CCNB1;NCAPG |
| progesterone CTD 00006624 | 0.001072613 | 0.043023718 | CXCL10;CCNB1;NCAPG;IL7R |
| 1,9-Pyrazoloanthrone CTD 00003948 | 0.001192912 | 0.043064119 | CXCL10;CCNB1 |
| Demecolcine CTD 00005762 | 0.001516156 | 0.044550912 | CXCL10;CCNB1;IL7R |
| Silica CTD 00006678 | 0.001629261 | 0.044550912 | CXCL10;NCAPG;IL7R |
| PHENCYCLIDINE CTD 00005881 | 0.001850684 | 0.044550912 | CXCL10;CCNB1 |
| vinblastine CTD 00006986 | 0.001866885 | 0.044550912 | CCNB1;NCAPG |
| 1-chloro-2,4-dinitrobenzene CTD 00005848 | 0.002118083 | 0.044550912 | CXCL10;IL7R |
| Allococaine CTD 00005697 | 0.003024211 | 0.044550912 | CXCL10;PRTN3 |
| CHEMBL380598 TTD 00005159 | 0.003295826 | 0.044550912 | CCNB1 |
| Fulvestrant CTD 00002740 | 0.003447238 | 0.044550912 | CXCL10;CCNB1 |
| AR-A014418 CTD 00004251 | 0.003594998 | 0.044550912 | CCNB1 |
| 3-NITROFLUORANTHENE CTD 00001617 | 0.003594998 | 0.044550912 | CXCL10 |
| DENSPM CTD 00002486 | 0.003894096 | 0.044550912 | CCNB1 |
| roscovitine TTD 00010683 | 0.003894096 | 0.044550912 | CCNB1 |
| 1H-Pyrrole-2,5-dione, 3,4-diphenyl- TTD 00000418 | 0.003894096 | 0.044550912 | CCNB1 |
| 1H-Pyrrole-2,5-dione, 3,4-bis(4-methoxyphenyl)- TTD 00000417 | 0.003894096 | 0.044550912 | CCNB1 |
| Trabectedin CTD 00003298 | 0.003894096 | 0.044550912 | CCNB1 |
| AGN-PC-00BUNE TTD 00001660 | 0.003894096 | 0.044550912 | CCNB1 |
| Roflumilast CTD 00003916 | 0.003894096 | 0.044550912 | CXCL10 |
| indomethacin CTD 00006147 | 0.004013148 | 0.044550912 | CXCL10;CCNB1 |
| pd 168393 CTD 00004570 | 0.004492068 | 0.044550912 | CXCL10 |
| rolipram CTD 00007371 | 0.004492068 | 0.044550912 | CXCL10 |
| Homocastasterone CTD 00002741 | 0.004492068 | 0.044550912 | CCNB1 |
| Rimonabant hydrochloride CTD 00003133 | 0.004790942 | 0.044550912 | CCNB1 |
| Brassinolide CTD 00001097 | 0.004790942 | 0.044550912 | CCNB1 |
| Calpain inhibitor I CTD 00002578 | 0.005089741 | 0.044550912 | CCNB1 |
| allopurinol CTD 00005353 | 0.005089741 | 0.044550912 | CXCL10 |
| mechlorethamine CTD 00006251 | 0.005388466 | 0.044550912 | CCNB1 |
| Tylophorine CTD 00000134 | 0.005388466 | 0.044550912 | CCNB1 |
| Methoxycamptothecin CTD 00001213 | 0.005687116 | 0.044550912 | CCNB1 |
| deferoxamine MCF7 DOWN | 0.005985691 | 0.044550912 | CCNB1 |
| POTASSIUM BROMATE CTD 00000989 | 0.006284191 | 0.044550912 | CCNB1 |
| Polydatin CTD 00002437 | 0.006284191 | 0.044550912 | CXCL10 |
| monobenzone MCF7 DOWN | 0.006284191 | 0.044550912 | CCNB1 |
| AFLATOXIN B1 CTD 00007128 | 0.006490318 | 0.044550912 | CXCL10;CCNB1;NCAPG;PRTN3 |
| sodium azide CTD 00007311 | 0.006582617 | 0.044550912 | CCNB1 |
| triprolidine PC3 UP | 0.006582617 | 0.044550912 | IL7R |
| IN1541 CTD 00001481 | 0.006880968 | 0.044550912 | CCNB1 |
| Adenylyl sulfate BOSS | 0.007179245 | 0.044550912 | DEFA4 |
| Cryptolepine CTD 00001119 | 0.007179245 | 0.044550912 | CCNB1 |
| 2,6-DICHLOROINDOPHENOL CTD 00007030 | 0.007179245 | 0.044550912 | CXCL10 |
| lobeline HL60 DOWN | 0.007220608 | 0.044550912 | CCNB1;NCAPG;PRTN3 |
| apicidin CTD 00003381 | 0.007477447 | 0.044550912 | CCNB1 |
| Bis-gma CTD 00007186 | 0.007775574 | 0.044550912 | CCNB1 |
| Antimycin A CTD 00005427 | 0.007775574 | 0.044550912 | CCNB1 |
| bisindolylmaleimide i TTD 00002466 | 0.008073627 | 0.044550912 | CCNB1 |
| alexidine HL60 DOWN | 0.008073627 | 0.044550912 | PRTN3 |
| roscovitine CTD 00003426 | 0.008371605 | 0.044550912 | CCNB1 |
| paclitaxel CTD 00007144 | 0.008516845 | 0.044550912 | CCNB1;NCAPG |
| kenpaullone TTD 00008780 | 0.008669509 | 0.044550912 | CCNB1 |
| D-MONAPTERIN BOSS | 0.008669509 | 0.044550912 | CXCL10 |
| 2-methoxy-13-methyl-6,7,8,9,11,12,14,15,16,17-decahydrocyclopenta[a]phenanthrene-3,17-diol CTD 00001063 | 0.008669509 | 0.044550912 | CCNB1 |
| bisindolylmaleimide iv TTD 00002467 | 0.008669509 | 0.044550912 | CCNB1 |
| mefloquine HL60 UP | 0.008669509 | 0.044550912 | CXCL10 |
| Dasatinib CTD 00004330 | 0.008820266 | 0.044550912 | CXCL10;NCAPG |
| clindamycin HL60 DOWN | 0.008894018 | 0.044550912 | CCNB1;NCAPG;PRTN3 |
| chlorophyllin CTD 00000324 | 0.008967338 | 0.044550912 | CXCL10 |
| monorden PC3 UP | 0.008967338 | 0.044550912 | IL7R |
| gsno CTD 00007396 | 0.009265092 | 0.044550912 | CXCL10 |
| Cobalt sulfate CTD 00001238 | 0.009562772 | 0.044550912 | IL7R |
| minocycline CTD 00006338 | 0.009562772 | 0.044550912 | PRTN3 |
| 3-Aminofluoranthene CTD 00002071 | 0.009860378 | 0.044550912 | CXCL10 |
| Pemetrexed CTD 00003054 | 0.009860378 | 0.044550912 | IL7R |
| trifluridine MCF7 DOWN | 0.009860378 | 0.044550912 | CCNB1 |
| EGTA CTD 00005889 | 0.009860378 | 0.044550912 | CXCL10 |
| Riboflavine CTD 00006698 | 0.009860378 | 0.044550912 | CCNB1 |
| COPPER CTD 00005706 | 0.009878335 | 0.044550912 | CCNB1;NCAPG;IL7R |
| promethazine PC3 UP | 0.010157909 | 0.044550912 | IL7R |
| amsacrine CTD 00005396 | 0.010455365 | 0.044550912 | CCNB1 |
| pyrantel HL60 DOWN | 0.010455365 | 0.044550912 | PRTN3 |
| Go 6976 CTD 00002962 | 0.010455365 | 0.044550912 | CCNB1 |
| piroxicam CTD 00006571 | 0.01048436 | 0.044550912 | CCNB1;NCAPG |
| dirithromycin HL60 DOWN | 0.010743526 | 0.044550912 | NCAPG;PRTN3 |
| ciprofloxacin CTD 00005674 | 0.010752747 | 0.044550912 | CCNB1 |
| aspirin CTD 00005447 | 0.010930426 | 0.044550912 | CCNB1;IL7R |
| Romidepsin CTD 00003096 | 0.011050055 | 0.044550912 | CCNB1 |
| prenylamine HL60 UP | 0.011347288 | 0.044550912 | CXCL10 |
| usnic acid CTD 00002807 | 0.011347288 | 0.044550912 | CXCL10 |
| 4-Aminobenzohydrazide CTD 00001642 | 0.011347288 | 0.044550912 | PRTN3 |
| NICKEL SULFATE CTD 00001417 | 0.011499996 | 0.044550912 | CXCL10;IL7R |
| pentoxifylline CTD 00006487 | 0.011644447 | 0.044550912 | CCNB1 |
| Calycosin CTD 00003727 | 0.011644447 | 0.044550912 | CCNB1 |
| Decitabine CTD 00000750 | 0.011820492 | 0.044550912 | CXCL10;CCNB1;IL7R |
| RUTIN CTD 00006712 | 0.012238541 | 0.044550912 | CXCL10 |
| TETRACHLOROETHYLENE CTD 00006849 | 0.012238541 | 0.044550912 | NCAPG |
| mephentermine HL60 DOWN | 0.012238541 | 0.044550912 | CCNB1 |
| LY-294002 PC3 DOWN | 0.012238541 | 0.044550912 | IL7R |
| DIALLYL DISULFIDE CTD 00001321 | 0.012535477 | 0.044550912 | CCNB1 |
| Allyl sulfide CTD 00001796 | 0.012535477 | 0.044550912 | CCNB1 |
| Acteoside CTD 00002463 | 0.012535477 | 0.044550912 | CXCL10 |
| 0173570-0000 PC3 DOWN | 0.012832338 | 0.044550912 | CCNB1 |
| Gadodiamide hydrate CTD 00002623 | 0.012832338 | 0.044550912 | CXCL10 |
| Acetovanillone CTD 00002374 | 0.013129125 | 0.044550912 | CXCL10 |
| oxolinic acid HL60 DOWN | 0.013129125 | 0.044550912 | PRTN3 |
| dorsomorphin CTD 00004649 | 0.013425837 | 0.044550912 | CCNB1 |
| flurbiprofen CTD 00005993 | 0.013425837 | 0.044550912 | CCNB1 |
| 2-CHLOROETHYL ETHYL SULFIDE CTD 00001489 | 0.013425837 | 0.044550912 | CCNB1 |
| POTASSIUM CHROMATE CTD 00001284 | 0.013677898 | 0.044550912 | CXCL10;CCNB1;IL7R |
| geldanamycin PC3 UP | 0.013722475 | 0.044550912 | IL7R |
| Inosinic acid BOSS | 0.013722475 | 0.044550912 | CXCL10 |
| AMILORIDE CTD 00005369 | 0.014019039 | 0.044550912 | CCNB1 |
| mitotane CTD 00006344 | 0.014019039 | 0.044550912 | CCNB1 |
| loxapine PC3 UP | 0.014019039 | 0.044550912 | IL7R |
| 2-Mercaptobenzothiazole CTD 00000240 | 0.014019039 | 0.044550912 | IL7R |
| Cardidigin CTD 00005822 | 0.014019039 | 0.044550912 | CCNB1 |
| pizotifen PC3 UP | 0.014315529 | 0.044550912 | IL7R |
| gossypol HL60 DOWN | 0.014315529 | 0.044550912 | PRTN3 |
| nocodazole BOSS | 0.014315529 | 0.044550912 | CCNB1 |
| 8-Bromo-cAMP, Na CTD 00007044 | 0.014587441 | 0.045009113 | CXCL10;IL7R |
| Leptomycin B CTD 00001805 | 0.014908285 | 0.045296077 | CXCL10 |
| calcitriol CTD 00005558 | 0.014931394 | 0.045296077 | CCNB1;NCAPG;IL7R |
| methotrexate MCF7 DOWN | 0.015500744 | 0.046631405 | CCNB1 |
| raloxifene CTD 00007367 | 0.016075672 | 0.047619174 | CCNB1;IL7R |
| Picrotoxinum BOSS | 0.016092906 | 0.047619174 | CCNB1 |
| Fisetin CTD 00000919 | 0.016684772 | 0.048969127 | CCNB1 |
| FERROUS SULFATE CTD 00001009 | 0.016980593 | 0.049435437 | CXCL10 |
| nocodazole CTD 00007087 | 0.017276341 | 0.049498087 | CCNB1 |
| Alvocidib CTD 00002892 | 0.017276341 | 0.049498087 | CCNB1 |
| Caffeic acid phenethyl ester CTD 00002352 | 0.017572014 | 0.049948796 | CCNB1 |
