# Supplementary material for: Identification and validation of diagnostic markers and drugs for pediatric bronchopulmonary dysplasia based on integrating bioinformatics and molecular docking analysis
Source: PLoS One. 2025 May 7;20(5):e0323006. doi: 10.1371/journal.pone.0323006 (PMC12057968; doi:10.1371/journal.pone.0323006)
Supplement: S1 Fig — (DOCX) [file pone.0323006.s006.docx]

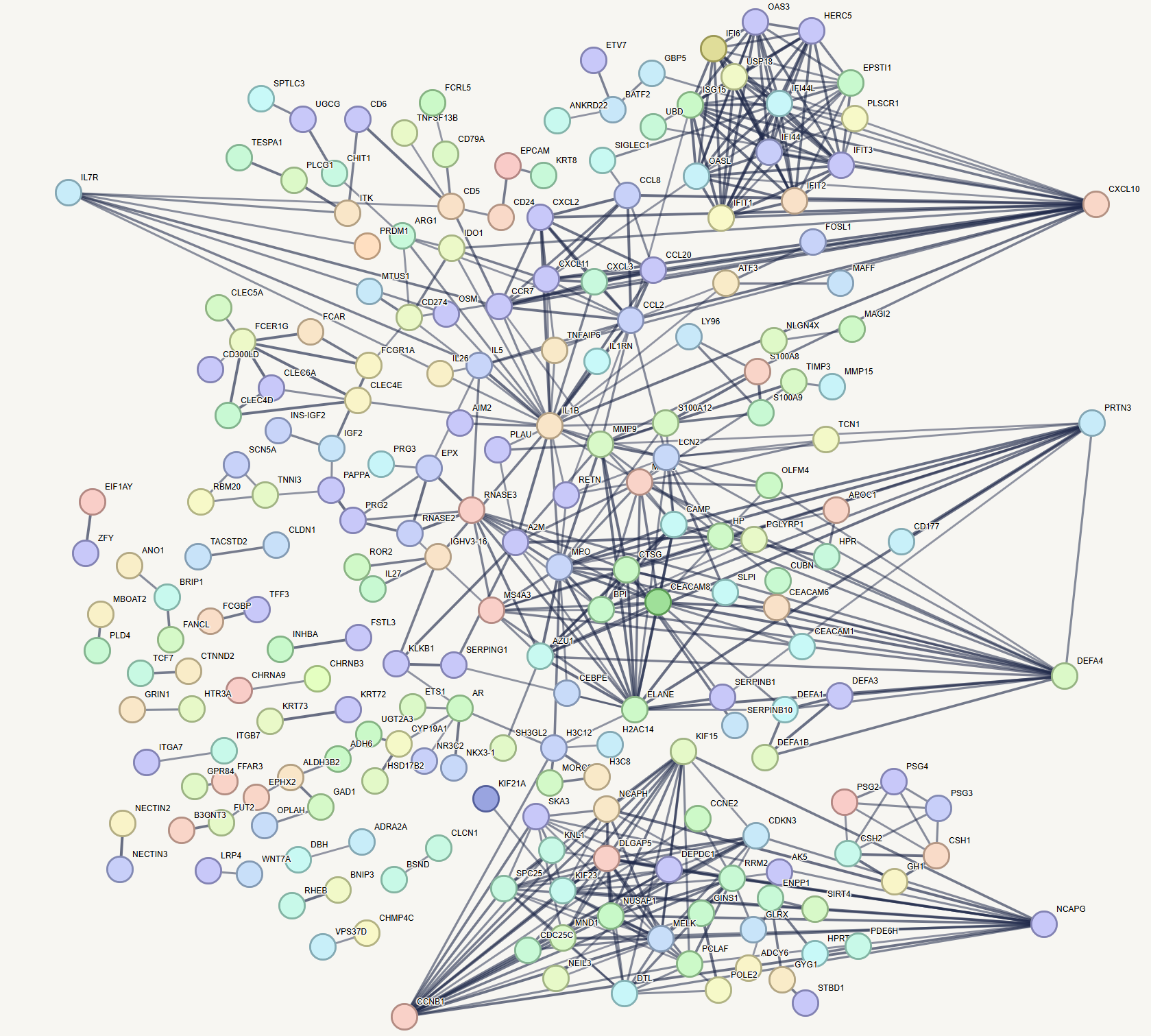


S1 Fig. The PPI network via STRING database. The nodes without connections with others were hidden. The red circle indicated the final optimal hub genes(IR7R, CXCL10, PRTN3, DEFA4, CCNB1, NCAPG) .Abbreviations:PPI,protein-protein interaction; STRING,the Search Tool for the Retrieval of Interacting Genes;IL7R, Interleukin-7 receptor, DEFA4, Defensin Alpha4 gene or Neutrophil Peptide 4, CCNB1, Cyclin B1, NCAPG, Non-SMC condensin I complex subunit G, CXCL10, C-X-C motif chemokine 10, PRTN3, Proteinase 3.
