# Supplementary figures and images for: Identification and validation of diagnostic markers and drugs for pediatric bronchopulmonary dysplasia based on integrating bioinformatics and molecular docking analysis
Source: PLoS One. 2025 May 7;20(5):e0323006. doi: 10.1371/journal.pone.0323006 (PMC12057968; doi:10.1371/journal.pone.0323006)

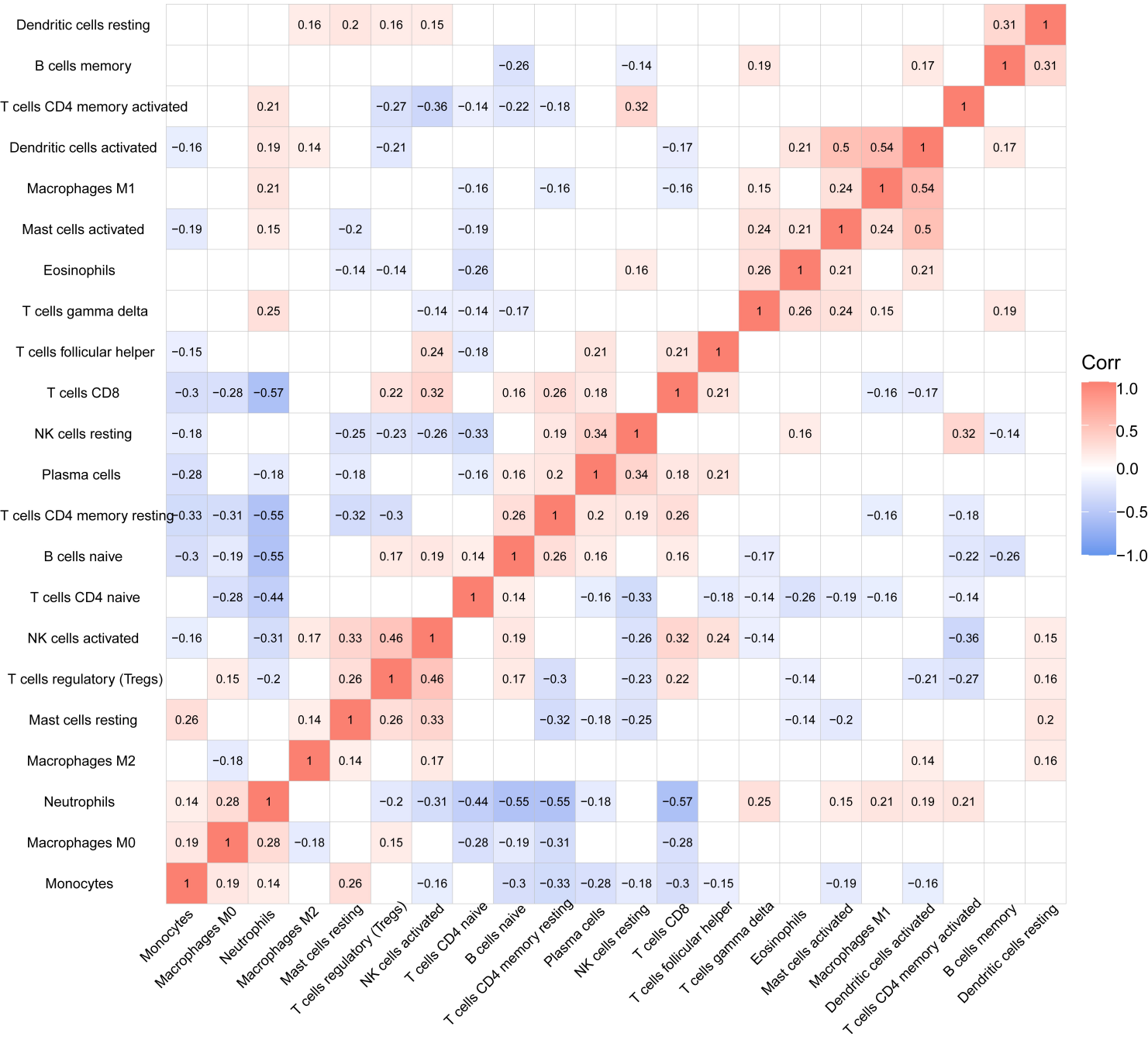


S2 Fig The correlated heatmap showed the correlation analysis of immune infiltrated cells.

Supplement: S2 Fig — (DOCX) [file pone.0323006.s007.docx]
