# Supplementary material for: Identification and validation of diagnostic markers and drugs for pediatric bronchopulmonary dysplasia based on integrating bioinformatics and molecular docking analysis
Source: PLoS One. 2025 May 7;20(5):e0323006. doi: 10.1371/journal.pone.0323006 (PMC12057968; doi:10.1371/journal.pone.0323006)
Supplement: S3 Fig — (DOCX) [file pone.0323006.s008.docx]

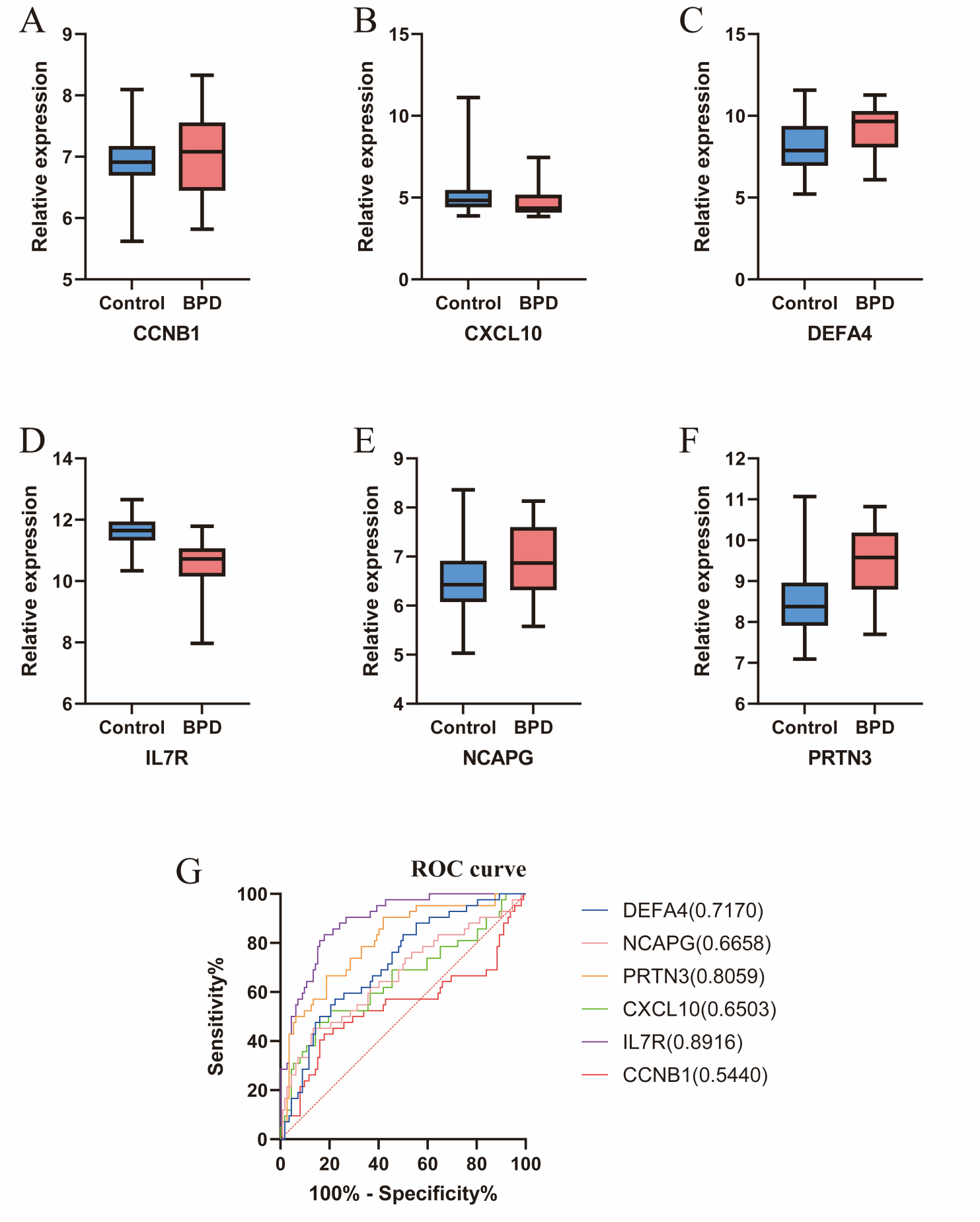


S3 Fig. The diagnostic value evaluation and nomogram construction of the validation cohort. (A-F)The Box plot showed expression of hub genes in BPDs and non-BPD groups. (G) The ROC curve of 6 hub genes in BPD. The number in the parentheses represents the AUC (Area Under the Curve).
